# Structural basis for a complex I mutation that blocks pathological ROS production

**DOI:** 10.1038/s41467-021-20942-w

**Published:** 2021-01-29

**Authors:** Zhan Yin, Nils Burger, Duvaraka Kula-Alwar, Dunja Aksentijević, Hannah R. Bridges, Hiran A. Prag, Daniel N. Grba, Carlo Viscomi, Andrew M. James, Amin Mottahedin, Thomas Krieg, Michael P. Murphy, Judy Hirst

**Affiliations:** 1 MRC Mitochondrial Biology Unit, University of Cambridge, Cambridge Biomedical Campus, Cambridge, UK; 2 Department of Medicine, University of Cambridge, Cambridge, UK; 3 William Harvey Research Institute, Barts and The London School of Medicine and Dentistry, Queen Mary University of London, London, UK; 4 Centre for Inflammation and Therapeutic Innovation, Queen Mary University of London, London, UK; 5 Department of Physiology, Institute of Neuroscience and Physiology, Sahlgrenska Academy, University of Gothenburg, Gothenburg, Sweden; 6 Present Address: Department of Biomedical Sciences, University of Padova via Ugo Bassi 58/B, Padova, 35131 Italy

**Keywords:** Biophysical chemistry, Cryoelectron microscopy, Bioenergetics

## Abstract

Mitochondrial complex I is central to the pathological reactive oxygen species (ROS) production that underlies cardiac ischemia–reperfusion (IR) injury. ND6-P25L mice are homoplasmic for a disease-causing mtDNA point mutation encoding the P25L substitution in the ND6 subunit of complex I. The cryo-EM structure of ND6-P25L complex I revealed subtle structural changes that facilitate rapid conversion to the “deactive” state, usually formed only after prolonged inactivity. Despite its tendency to adopt the “deactive” state, the mutant complex is fully active for NADH oxidation, but cannot generate ROS by reverse electron transfer (RET). ND6-P25L mitochondria function normally, except for their lack of RET ROS production, and ND6-P25L mice are protected against cardiac IR injury in vivo. Thus, this single point mutation in complex I, which does not affect oxidative phosphorylation but renders the complex unable to catalyse RET, demonstrates the pathological role of ROS production by RET during IR injury.

## Abstract (web summary)

Reactive oxygen species (ROS) production by reverse electron transfer (RET) through complex I is thought to cause tissue damage from heart attacks. Here, the authors combine in vivo work with biochemical and cryo-EM analyses to characterize the effects of a P25L mutation in the ND6 subunit of mitochondrial complex I. They observe that this mutation does not affect oxidative phosphorylation but renders complex I unable to generate ROS by RET: ND6-P25L mice are protected against cardiac ischaemia–reperfusion injury, thus providing evidence for the proposed role of ROS production in myocardial infarction.

## Introduction

**Fig. 1:**
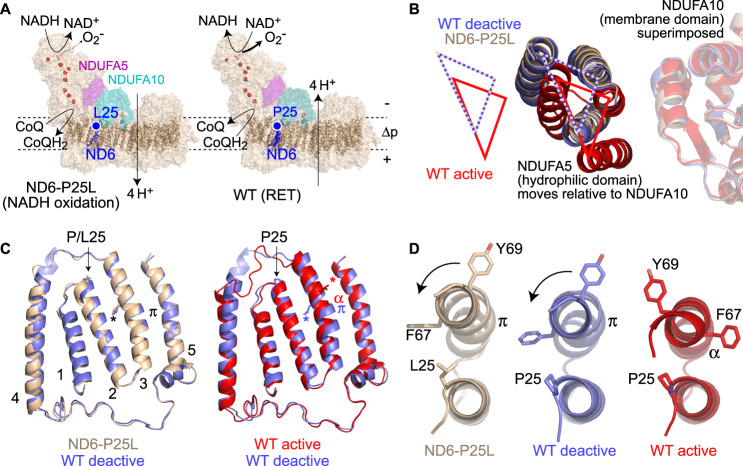
Structure of complex I containing the ND6-P25L mutation. **A** The NADH oxidation reaction is shown on the structure of ND6-P25L complex I (left). Complex I oxidizes NADH and reduces CoQ, and pumps four protons out of the mitochondrion to support the proton motive force (Δp). The reverse electron transfer (RET) reaction is shown on the structure of WT complex I (right). When Δp is large and the CoQ pool is highly reduced, electron and proton transfer at complex I are reversed; CoQ is oxidized and NAD^+^ reduced to NADH. Alternatively, the electrons are passed to O_2_ to form superoxide (O_2_^−^). The site of the mutation and ND6 subunit are shown, along with subunits NDUFA5 and NDUFA10. **B** The relative orientation of the NDUFA10 and NDUFA5 subunits on the hydrophilic and membrane arms, respectively (see **A**), defines ND6-P25L-CI to be in the deactive state. With the three structures superimposed by NDUFA10, the position of the three-helix bundle of NDUFA5 clearly differentiates the active and deactive states. **C** Comparison of the structures of the ND6 subunit in the WT-D and ND6-P25L complexes (left) and the WT-A and WT-D complexes (right). The gap between the top of TMHs 2 and 3 opens in ND6-P25L relative to WT-D, whereas the π-bulge is present in the WT-D and ND6-P25L complexes but not in WT-A. The asterisks mark the sidechain of ND6-Leu64 to visualize the rotation of the upper part of ND6 TMH3. Structures superimposed on the adjacent ND4L subunit. **D** Views from above TMHs 2 and 3 showing the rotation in the upper section of TMH3 (carrying Phe67 and Tyr69, top) that must occur to convert the WT-D and ND6-P25L complexes to the active state, and the decreasing (left to right) gap between them. Structures aligned to TMH2 (bottom). See also Supplementary Figs. 1–4, and Supplementary Tables 1 and 2.

Mitochondrial complex I catalyses the first step in the mammalian respiratory chain, electron transfer from NADH to CoQ (ubiquinone) coupled to proton transfer across the mitochondrial inner membrane, in order to generate the proton motive force (Δp) and drive ATP synthesis by oxidative phosphorylation. This asymmetric ~1 MDa complex comprises 45 protein subunits, encoded on both the mitochondrial and nuclear genomes, plus a flavin mononucleotide (FMN) and eight iron sulfur (FeS) centers to connect the active site for NADH oxidation from the matrix to the site of CoQ reduction from the membrane^1–3^ (Fig. 1A). Mutations to either the nuclear or mitochondrial (mtDNA) encoded subunits of complex I lead to devastating neuromuscular disorders that are typically associated with disrupted complex I catalysis and decreased mitochondrial ATP production^4^.

The *ND6* G14600A mtDNA mutation that leads to a proline to leucine substitution at position 25 in the ND6 subunit of complex I (ND6-P25L) was first discovered in a patient homoplasmic for the mutation who presented with Leigh syndrome and sensorineural deafness and died at 8 months of age^5^. The mutation was confirmed to cause an isolated complex I deficiency in muscle tissue and fibroblasts, and the effects were replicated in human transmitochondrial cybrids, which displayed hardly any complex I activity and drastically decreased amounts of the fully assembled enzyme^5^. However, a homoplasmic mouse model containing the corresponding *ND6* G13997A mtDNA mutation, which also causes the ND6-P25L substitution, exhibited far milder effects and was presented as a model for Leber’s Hereditary Optic Neuropathy^6^. Varying decreases in complex I-linked activity (by 20–60%) were reported in synaptosomes and heart and liver mitochondria, but neither the amount of the complex present in tissues, nor the ATP levels in synaptosomes appeared to be affected^6,7^. Intriguingly, although the mechanism remained unclear, mitochondria from the ND6-P25L variant exhibited a clear absence of reactive oxygen species (ROS) production by reverse electron transfer (RET) through complex I, driven by succinate oxidation^6,7^. In comparison, the ROS produced by NADH-linked substrates showed only small and variable increases compared to the wild-type enzyme.

When Δp is high and the CoQ pool is adequately reduced, the driving force for reverse proton transfer across the membrane is sufficient to drive electrons backwards through complex I, from reduced CoQ (CoQH_2_) to the flavin (Fig. 1A). This reversal of the normal electron transfer reaction causes a substantial increase in complex I-mediated production of superoxide, which is then dismutated to H_2_O_2_ by Mn-superoxide dismutase in the matrix^8,9^, and termed here “ROS production by RET”. Mitochondrial ROS production by RET has been directly implicated in redox signaling in inflammation^10^, oxygen sensing^11,12^, activation of uncoupling by mitochondria in brown adipose tissue^13,14^, and in aging and the stress response in flies^15,16^. Furthermore, mitochondrial ROS production contributes to the tissue damage associated with cardiac ischemia–reperfusion (IR) injury^17–19^. However, much of the pathological and physiological significance of RET through complex I in vivo remains unclear, and the mechanism of ROS production by RET remains contentious. A variant of mammalian complex I that is unable to catalyse RET thus provides a wealth of opportunities for pathological, physiological and mechanistic insights.

Here, we began by determining the structure of the ND6-P25L variant of complex I from homoplasmic mice. Surprisingly, our structure showed the complex to be predominantly in the ‘deactive’ (D) state, a pronounced resting state that usually forms only very slowly, when the complex is not actively turning over, that can be rapidly reactivated to the ‘active’ (A) state by the addition of substrates^20–23^. In contrast, matching preparations of wild-type (WT) mouse complex I are predominantly in the A state, and require a prolonged warm incubation under non-turnover conditions for conversion to the D state^22^. Analyses of the structure revealed how subtle perturbations at the site of the mutation propagate through the protein to the CoQ-binding site, which is disordered in the D state, and kinetic analyses showed that the variant complex exhibits D-like characteristics even during turnover. Because the conditions for ‘forward’ catalysis (NADH, CoQ and low Δp) efficiently return the D state enzyme to A status, but those for reverse catalysis (NAD^+^, CoQH_2_ and high Δp) are unable to do so, the rate of NADH oxidation by the ND6-P25L variant closely matches that of WT, but the rate of RET by the variant is negligible. This structural and molecular basis for the lack of RET by the ND6-P25L variant of complex I in vitro prompted us to investigate the proposed pathological role of mitochondrial ROS production by RET in cardiac IR injury in vivo.

We also show that the ND6-P25L mice, which are unable to catalyze RET, are protected against cardiac IR injury in vivo, confirming the pathological role of mitochondrial ROS production by RET through complex I in heart attacks.

## Results

### Structure of ND6-P25L complex I in the deactive state

To determine how the ND6-P25L mutation affects the structure of complex I, the complex (ND6-P25L-CI) was isolated from the heart mitochondria of ND6-P25L mice, and its structure determined by single-particle electron cryomicroscopy (cryo-EM). The catalytic (NADH:decylubiquinone oxidoreductase) activity of the preparation was 7.8 ± 0.4 (mean ± S.E.M., *n* = 3) μmol min^−1^ mg^−1^, relative to 10–12 μmol min^−1^ mg^−1^ for previously characterized preparations of WT mouse complex I (WT-CI)^22^, reflecting a modest loss of either specific activity or stability. The final cryo-EM density map reached a global resolution of 3.8 Å from 26,638 particles in the final reconstruction (Supplementary Figs. 1, 2, and Supplementary Table 1). The map revealed a fully intact complex matching WT-CI with all 45 subunits clearly present (Fig. 1A) and was described using a model containing 8063 residues (96% of the total, Supplementary Table 2) developed from models for the WT enzyme^22^.

Comparison of the map and model for ND6-P25L-CI with published maps and models for the A and D states of WT-CI showed clearly that it is in the D state. First, in WT-CI, the A and D complexes differ globally through the relative arrangement of their hydrophilic and membrane domains. The map-to-map correlation with the D state (EMDB-4356, 3.9 Å resolution) was 91–92%, relative to 78–82% with the A state (EMDB-11377, filtered to 3.9 Å resolution). Furthermore, the relative positions of two subunits, NDUFA10 on the membrane domain and NDUFA5 on the hydrophilic domain, provide a clear visual definition of the A/D transition, during which the two domains move relative to each other^22^, confirming ND6-P25L-CI is in the D state (Fig. 1B). Closer inspection of the ND6-P25L-CI model revealed all the known hallmarks of the D state^2,22,23^, particularly the π-bulge in TMH3 of the ND6 subunit (Fig. 1C and Supplementary Fig. 3) and extensive disorder of the loops in subunits ND1, ND3, and NDUFS2 that form parts of the CoQ-binding site (Supplementary Fig. 4). In stark contrast, WT-CI, prepared by the same method, is observed predominantly in the A state, with a 30-min, substrate-free incubation at 37 ˚C in vitro required for deactivation^22^.

To better compare the structures of ND6-P25L-CI and deactive WT-CI (WT-D), we reprocessed previously described cryo-EM data for the D state with updated software, obtaining a substantial improvement in resolution from 3.9 to 3.2 Å, and updated the model accordingly (Supplementary Figs. 1, 2, and Supplementary Tables 1, 2). The ND6-P25L-CI and WT-D structures match very closely overall (RMSD value 0.59 Å) and reveal only subtle structural differences in the vicinity of the mutation itself (Fig. 1C). The variant residue is located at the very start of ND6-TMH2 (on the matrix side of the membrane), pointing towards ND6-TMH3. In ND6-P25L-CI, TMH2 leans away from TMH3, relative to in WT-D (and WT-A) (Fig. 1C), increasing the distance between them (Fig. 1D and Supplementary Fig. 3). Most likely this occurs because of destabilizing steric interactions between the variant Leu sidechain on ND6-TMH2 and residues at the end of ND6-TMH3 and the start of the adjacent ND4L-TMH2, notably ND4L-Met27. As the P25L position sits closer to ND6-TMH3 in WT-A than WT-D (Fig. 1D), clashing particularly with ND6-Thr70, the A state of ND6-P25L-CI may be more strongly affected or destabilized than is visualized in our deactive structure.

ND6-TMH3 is important in the A/D transition as it contains a central π-bulge in the D state, but is α-helical throughout in the A state^22^. π-bulges are short sections of π-helix, less tightly wound than the standard α-helix. When the π-bulge is formed the upper (matrix) part of TMH3 rotates, relaxing the helical turn; conversely, destruction of the π-bulge during activation tightens the helical turn (Fig. 1C, D). Notably, the upper part of TMH3 contains two bulky aromatic residues, Phe67 and Tyr69, which adopt different rotational positions in the two states. Crucially, Phe67 transits through the TMH3–TMH2 interface, from one side to the other, as the upper part of TMH3 rotates (Fig. 1D and Supplementary Movie 1). Steric hindrance to the rotation, from these and other residues, likely explains why deactivation is so slow in the WT enzyme. Conversely, in ND6-P25L-CI, Leu25 pushes TMH2 away from the upper part of TMH3, decreasing the steric hindrance for the rotation, thereby accelerating deactivation by decreasing the energy barrier (Fig. 1D and Supplementary Movie 1). The ND6 TMH3–4 loop, which arches over the top of TMH2, also responds to deactivation (Fig. 1C). Comparison of structural data from the enzymes of different species has shown recently^24^ how formation of the π-bulge turns the top of ND6-TMH3 and rotates Phe67 away from the tight packing contact it makes with ND1, altering the conformation of ND1-TMH4, and relocating ND1-Tyr127 (at the top of ND1-TMH4) away from ND3-Cys39. As a result, the ND3-TMH1–2 loop that carries Cys39 is no longer anchored and becomes disordered, exposing the Cys to derivatization and NDUFS2 and NDUFS7 loops in the CoQ-binding site to the matrix. The resulting loss of structural integrity in the CoQ-binding site^2,22,23^ explains why the D state is catalytically inactive. Our structures thus reveal how the subtle ND6-P25L mutation destabilizes the CoQ-binding site, and indicate why spontaneous conversion of ND6-P25L-CI to the more stable D state happens far more rapidly than in WT-CI—consistent with the absence of the A state from our cryo-EM analyses of the mutant enzyme.

### ND6-P25L complex I is active but with deactive-like characteristics

**Fig. 2:**
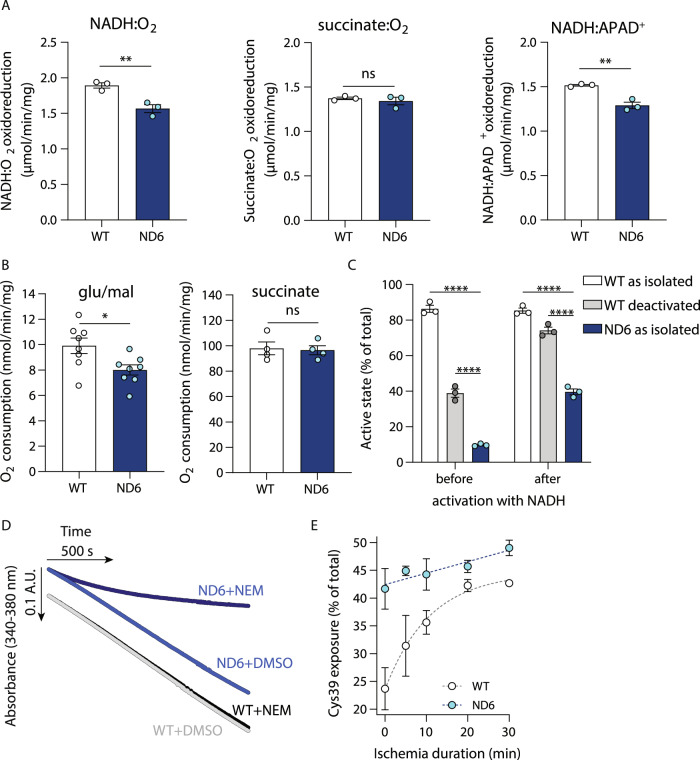
The active/deactive status and catalytic activity of ND6-P25L and WT complex I. **A** The rates of NADH and succinate oxidation by complexes I–III–IV and II–III–IV, respectively, in mitochondrial membranes from WT and ND6-P25L (referred to as ND6) mouse heart tissue, and the rate of the complex I-specific NADH:APAD^+^ oxidoreduction reaction. The data are mean averages ± S.E.M. (*n* = 3, from three independent preparations each comprising four or five hearts) evaluated using an unpaired, two-tailed Student’s *t*-test (**p < 0.01; *p* values are 0.0083, 0.5347, 0.0036, respectively). The decreased rate of NADH oxidation in ND6-P25L (83% of WT) is due to the lower amount of complex I present (85% of WT in the NADH:APAD^+^ assay). **B** The rates of O_2_ consumption by isolated heart mitochondria during respiration on glutamate/malate (0.5 mM) and upon subsequent addition of succinate (10 mM). Mitochondria were isolated from four hearts and pooled to provide a single test sample. The data are mean averages ± S.E.M. (glu/mal (glutamate/malate), *n* = 8, succinate, *n* = 4, technical replicates) evaluated using an unpaired, two-tailed Student’s *t*-test (*p < 0.05; *p* values are 0.02 (glu/mal), 0.83 (succinate)). **C** The percentage of complex I in A-like states in ND6-P25L and WT membranes, either as-isolated or deactivated by incubation for 30 min at 37 °C in the absence of substrates, and before or after activation by addition of 1 mM NADH at room temperature for 10 s. The rates of NADH oxidation by samples treated with NEM were compared to the rates from NEM-free control samples to determine the proportion of A-like complex I present. The data are mean averages ± S.E.M. (*n* = 3, from three independent preparations each comprising four or five hearts), evaluated using a 2-way ANOVA test with Tukey’s multiple comparisons correction (****p < 0.0001). **D** Catalytic activity assays of NADH oxidation by mitochondrial membranes from WT and ND6-P25L mouse hearts show linear rates of catalysis. The membranes are fully active prior to addition of 1 mM NEM at the start of the traces shown here. The NEM does not affect catalysis by WT-CI, but rapidly inhibits catalysis by ND6-P25L-CI. **E** Deactivation of complex I determined by differential labeling of the ND3-Cys39 peptide using light and heavy (^13^C_2_, 2-d_2_) labeled iodoacetamide, followed by LC-MS/MS analysis. The data are the percentage of ND3-Cys39 that is exposed to derivatization at the start of tissue homogenization for hearts from WT and ND6-P25L mice, following various times of ischemia. The data are mean averages ± S.E.M. from experiments on three independent hearts.

Comparison of membranes prepared from ND6-P25L heart mitochondria with those from WT mitochondria (Fig. 2A) revealed a small decrease in NADH:O_2_ oxidoreduction rate (the NADH:O_2_ reaction) through complexes I, III and IV, whereas the succinate:O_2_ reaction (through complexes II, III and IV) was unchanged. The catalytic rates did not exhibit any substantial “lag phases” upon the addition of substrates. Similar results were observed between ND6-P25L and WT heart mitochondria respiring on the NADH-linked substrates glutamate and malate, or on succinate (Fig. 2B). The NADH:O_2_ reaction rate was normalized to the amount of complex I flavin site present by comparing it to the NADH:APAD^+^ oxidoreduction rate (Fig. 2A), revealing that the decreased activity is due to a lower content of ND6-P25L-CI than WT-CI, not to its lower specific activity, in contrast to previous reports^6,7^. Therefore, despite being structurally characterized in the D state, ND6-P25L-CI switches quickly and efficiently into a catalytically-active state that is as competent for NADH oxidation as WT-CI.

ND3-Cys39, on the ND3 TMH1–2 loop that becomes disordered in the D-state, is an important biochemical marker of the A/D status of complex I as it is exposed to thiol-derivatizing agents only in the D-state^20^. The susceptibility of ND3-Cys39 to alkylation by *N*-ethylmaleimide (NEM) was thus used to establish that, in the membrane preparations, ∼85% of WT-CI but only ∼10% of ND6-P25L-CI is NEM-insensitive, and therefore in an A-like state (Fig. 2C), consistent with the cryo-EM analyses. Subsequently, we attempted to observe ND6-P25L-CI in a predominantly NEM-insensitive, A-like state by applying pre-activation protocols. The complex was activated for 10 s by the addition of NADH at room temperature to induce turnover, then the membranes were treated with NEM on ice for 20 min, under which conditions both catalysis and the A–D transition are expected to occur very slowly. For ND6-P25L-CI the results revealed a mixture of states in which the A-like proportion of the population had increased, but still remained substantially below the levels observed starting from either WT-CI, or from WT-CI that had been pre-treated to deactivate it (Fig. 2C). Subsequently, it was found that in ND6-P25L-CI (but not WT-CI) ND3-Cys39 is exposed and able to react with NEM even when the complex is turning over and is thus in a catalytically-active state (Fig. 2D). These findings suggest that either: (i) while actively catalyzing NADH oxidation, ND6-P25L-CI leaves the catalytic cycle to transiently visit off-pathway states in which ND3-Cys39 is exposed; and/or (ii) the ND3-TMH1–2 loop is structurally altered and ND3-Cys39 exposed in one or more on-pathway states of ND6-P25L-CI during catalysis. In either case, the state(s) captured by NEM need not reflect full conversion of the enzyme to the structurally characterized, canonical D state. Notably, structures of complex I from the yeast *Yarrowia lipolytica* have revealed it to be in an ‘intermediate’ state, in which the corresponding π-bulge is present and ND3-Cys is exposed, but the CoQ-binding site remains relatively well-ordered^24^. This situation may resemble the states captured by NEM in ND6-P25L-CI.

To confirm that ND6-P25L-CI exhibits the same behavior in intact heart tissue, a semiquantitative differential cysteine labeling strategy, using light and heavy (^13^C_2_, 2-d_2_) iodoacetamide followed by LC-MS/MS-based detection of the labeled ND3-Cys39 tryptic peptide, was used to investigate the exposure of ND3-Cys39 in ischemic hearts (Fig. 2E). In WT hearts, the exposure of ND3-Cys39 increased gradually over time, reaching a plateau after 20 min ischemia, as described previously^25^. In contrast, in ND6-P25L hearts, ND3-Cys39 was already highly exposed in the first measurement, taken after the shortest period of ischemia possible. Although the level of exposure drifted upwards during further ischemia, even the initial level of Cys39 exposure for ND6-P25L hearts was comparable to that reached by WT hearts after 20–30 min ischemia. The much greater exposure of ND3-Cys39 in ND6-P25L-CI than WT-CI is thus clear within intact tissues as well as in the isolated complex.

### ND6-P25L complex I is unable to catalyze ROS production by RET

**Fig. 3:**
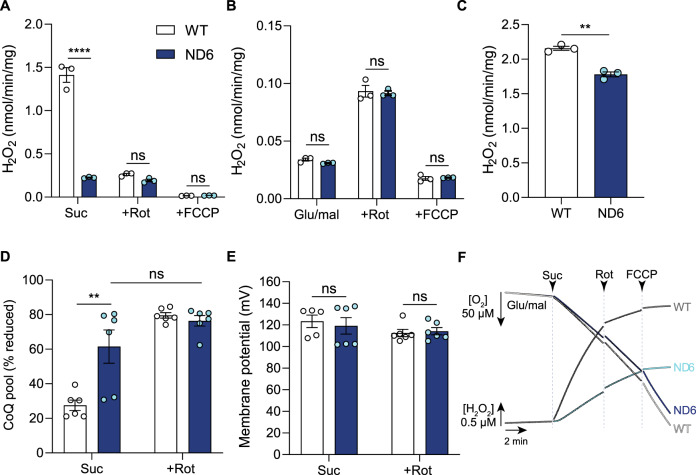
ND6-P25L complex I does not catalyze reverse electron transfer. Rates of production of H_2_O_2_ by isolated mitochondria from WT and ND6-P25L mouse hearts incubated with (**A**) succinate (10 mM) or (**B**) glu/mal (glutamate/malate; 10 mM of each). FCCP (5 µM) or rotenone (5 µM) were added as indicated. The data were obtained using a kinetic plate reader at room temperature and are mean averages ± S.E.M. (*n* = 3, where each replicate is from an independent mitochondrial preparation on a different heart) evaluated using a two-way ANOVA test with Tukey’s multiple comparisons correction (****p < 0.0001). **C** Production of H_2_O_2_ by mitochondrial membranes isolated from WT and ND6-P25L mouse hearts incubated with NADH in the presence of piericidin A. The data are mean averages ± S.E.M. (*n* = 3, from three independent preparations each comprising four or five hearts) evaluated using an unpaired, two-tailed Student’s *t*-test (**p = 0.0012). The decreased rate in ND6-P25L (83% of WT) is due to the lower amount of complex I present (85% of WT, Fig. 2A). **D** Redox status of the CoQ pool in isolated mitochondria from WT and ND6-P25L mouse hearts respiring on succinate. Rotenone (1 µM) was added as indicated. Data are reported for the predominant CoQ_9_ form. The data are mean averages ± S.E.M. (*n* = 6, where each replicate is from an independent mitochondrial preparation on a different heart). Statistical significance was assessed by a two-way ANOVA test with Tukey’s multiple comparisons correction (**p < 0.01; *p* values for WT vs ND6 suc, 0.0011; ND6 suc vs ND6 + Rot, 0.228). **E** Membrane potentials calculated from the accumulation of [^3^H]-TPMP by isolated mitochondria from WT and ND6-P25L mouse hearts respiring on succinate. Rotenone (0.5 µM) was added as indicated. The data are mean averages ± S.E.M. (WT-succinate, *n* = 5, all others *n* = 6, where each replicate is from an independent mitochondrial preparation on a different heart). The differences were assessed by a two-way ANOVA test with Tukey’s multiple comparisons correction. *P* values are 0.946 (Suc) and 0.996 (+Rot). **F** O_2_ consumption and H_2_O_2_ production by isolated heart mitochondria were measured at 37 °C in the O2K Oxygraph during respiration on glutamate/malate (0.5 mM). 10 mM succinate, 5 µM rotenone and 5 µM FCCP were added as indicated. A representative experiment is shown, typical of three replicates. See also Supplementary Fig. 5.

ROS production by RET through complex I was investigated in isolated heart mitochondria by using succinate oxidation to reduce the CoQ pool and generate Δp^8^. While WT mitochondria exhibited a substantial rate of H_2_O_2_ production that was sensitive to the complex I inhibitor rotenone, only very low rates of rotenone-sensitive activity were observed in ND6-P25L mitochondria (Fig. 3A). The rotenone-insensitive rates, which are not due to RET by complex I, were similar in both cases, and all the rates were sensitive to FCCP, which abolishes Δp. As the rate of succinate oxidation in both ND6-P25L mitochondrial membranes and mitochondria is normal (Fig. 2A and B) the results suggest that ND6-P25L complex I is unable to catalyze H_2_O_2_ production by RET, as discussed previously^6,7^. In contrast, the H_2_O_2_ production from ND6-P25L mitochondria respiring on the substrates glutamate and malate, where complex I catalyzes NADH oxidation and not RET, was similar to that from WT mitochondria, whether complex I was inhibited or not (Fig. 3B). While NADH-linked H_2_O_2_ production is lower in mitochondrial membranes from the ND6-P25L variant treated with the complex I inhibitor piericidin A (Fig. 3C), the decrease is to the same extent as that in the NADH:O_2_ and NADH:APAD^+^ reactions (Fig. 2A). Following normalization for the amount of enzyme present, the specific activities for NADH-linked H_2_O_2_ production are the same (ND6-P25L relative to WT: 97.2 ± 3.8%, mean ± S.E.M., *n* = 3). Therefore, only complex I ROS production by RET is affected by the ND6-P25L mutation.

To confirm that the lack of succinate-driven H_2_O_2_ production in ND6-P25L mitochondria is due to an intrinsic difference in complex I activity and not to a secondary effect on the thermodynamics across complex I, the CoQ redox status^26^ and Δp were quantified under RET conditions. The CoQ pool was more reduced in ND6-P25L mitochondria than WT mitochondria (Fig. 3D); that this is due to the lack of CoQH_2_ oxidation by RET through ND6-P25L-CI was confirmed by using rotenone to block RET in WT-CI, which equalized the CoQ redox states (Fig. 3D). The CoQ pool in both WT and ND6-P25L mitochondria is highly reduced in the presence of succinate and rotenone (Fig. 3D) and no significant differences were identified in the mitochondrial membrane potential (Δψ, the major component of Δp)^27^ (Fig. 3E). Therefore, the lack of ROS production by RET in ND6-P25L mitochondria is not due to a decrease in either of its thermodynamic drivers, relative to their values in WT mitochondria.

ND6-P25L-CI appears normal in every way, except that it is unable to catalyze RET and has a greater propensity than WT-CI to adopt deactive or deactive-like states, suggesting that these two unusual characteristics are related. Both ND6-P25L-CI and WT-D-CI can be activated to catalyze NADH oxidation upon the provision of NADH and CoQ. However, it is currently unclear whether ND6-P25L-CI is unable to catalyze RET because it collapses rapidly into D-like states that cannot be reactivated under RET conditions, or because it is intrinsically unable to catalyze RET, even when starting from states that are fully competent for NADH oxidation. Therefore, to switch ND6-P25L-CI rapidly from actively catalyzing NADH oxidation to conditions favoring RET, we used glutamate/malate to establish NADH:O_2_ oxidoreduction in mitochondria, then added succinate (Fig. 3F, Supplementary Fig. 5). For WT mitochondria, succinate induced substantial increases in both O_2_ consumption and H_2_O_2_ production, consistent with an immediate switch to RET catalysis. In contrast, for ND6-P25L mitochondria addition of succinate led to a matching increase in O_2_ consumption, but the increase in H_2_O_2_ production was both much smaller and fully insensitive to rotenone. Therefore, in contrast to WT-CI, ND6-P25L-CI is unable to switch on or sustain RET catalysis, regardless of the state it is in when RET is initiated.

### The ND6-P25L complex I mutation is protective against IR injury

Young adult mice homoplasmic for the ND6-P25L mutation are viable, they lack any gross phenotype and their complex I activity and ATP production are largely unimpaired^6,7^. Importantly, the hearts from ND6-P25L mice have been shown previously to be physiologically very similar to WT hearts by echocardiography^7^. Therefore, the lack of ROS production by RET through complex I in these mice defines an ideal model system to explore the postulated pathological role of ROS production by RET in IR injury^17,18^. During ischemia, lack of blood supply to the tissue causes O_2_ levels to drop, leading to a dramatic accumulation of mitochondrial succinate^18,28^. When the ischemic tissue is reperfused with oxygenated blood, for example upon release of an occluded coronary artery, the succinate is rapidly oxidized by the respiratory chain, driving RET through complex I, and the associated ROS formation is proposed to be a major cause of the tissue damage that occurs in heart attack, stroke, or organ transplantation^17,18,28^. If this model is correct, ND6-P25L mice should be protected against IR injury.

**Fig. 4:**
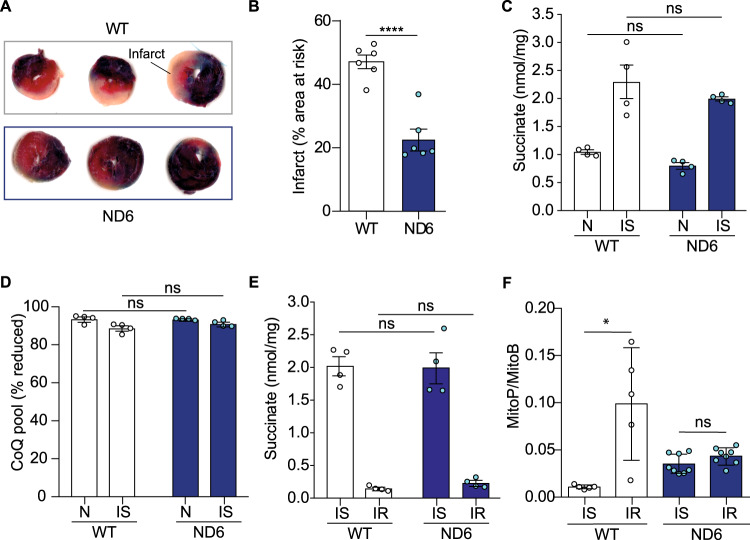
Homoplasmic ND6-P25L mice are protected against cardiac IR injury due to lack of ROS production by RET at complex I. **A** Representative images of slices from hearts showing cardiac infarcts, indicated by the light colored tissue, after 30 min of ischemia due to LAD occlusion, followed by 2 h reperfusion. **B** Quantification of cardiac infarct size as a proportion of the risk area in mice that underwent IR injury as in **A**. The data are mean averages ± S.E.M. (*n* = 6) evaluated using an unpaired, two-tailed Student’s *t*-test (****p < 0.0001, p = 6.4 ×10^−5^). **C**, **D** Accumulation of succinate (C) and CoQ (D) redox state during normoxia (N) and ischemia (IS) in heart tissue. Each heart was cut in half, one half frozen immediately and the other half exposed to ischemia for 30 min and then frozen. The data are mean averages ± S.E.M. (*n* = 4) assessed by a two-way ANOVA with Tukey’s multiple comparisons correction. **C** *P* values are 0.677 (N, WT vs ND6) and 0.538 (IS, WT vs ND6). **D** *P* values are 0.999 (N, WT vs ND6) and 0.320 (IS, WT vs ND6). **E** WT and ND6-P25L mouse hearts were Langendorff perfused before being subjected to either 20 min global ischemia (IS) or 20 min ischemia and 6 min reperfusion (IR); succinate levels were measured by LC-MS/MS. The data are mean averages ± S.E.M. (*n* = 4) evaluated by a two-way ANOVA with Tukey’s multiple comparisons correction. *P* values are 0.991 (IS, WT vs ND6) and 0.969 (IR, WT vs ND6). **F** Quantification of the MitoP/B ratio as a marker for mitochondrial H_2_O_2_ production. ND6-P25L and WT mice were injected with MitoB by the tail vein, and hearts exposed to either ischemia (IS, 25 min normoxia then 30 min ischemia); or ischemia and reperfusion (IR, 10 min normoxia then 30 min ischemia then 15 min reperfusion), total 55 min in each case. The data are mean averages ± S.E.M. (WT, *n* = 5; ND6, *n* = 8), evaluated using an unpaired, two-tailed Student’s *t*-test (*p < 0.05). The WT and ND6 cohorts were analyzed in separate experiments. *P* values are 0.0108 (WT, IS vs IR) and 0.104 (ND6, IS vs IR).

IR injury to the myocardium was compared in WT and ND6-P25L mice by occlusion of the left anterior descending (LAD) coronary artery, followed by reperfusion (Fig. 4A and B). Considerable damage to the heart was observed in WT mice but not in ND6-P25L mice, which are protected significantly against this form of cardiac IR injury (Fig. 4A and B). Importantly, the level of protection achieved against cardiac IR injury in the ND6-P25L mice was as great as that achieved by the most effective current therapeutic interventions such as ischemic preconditioning^29^. These findings are consistent with a central role for ROS production by complex I through RET in IR injury. During IR injury, RET at complex I is driven by succinate accumulation during ischemia and its subsequent oxidation upon reperfusion^17,18^, so we next assessed whether these processes were altered in the ND6-P25L mice. There were no significant differences in either normoxic or ischemic succinate levels (Fig. 4C), or CoQ redox state (Fig. 4D) between WT and ND6-P25L hearts. Furthermore, measurement of succinate levels in isolated Langendorff-perfused hearts exposed to global ischemia (Fig. 4E) further demonstrated that the ischemic accumulation of succinate was unaffected by the ND6-P25L mutation. Importantly, the succinate levels also decreased very rapidly and to similar extents in WT and ND6-P25L hearts upon reperfusion (Fig. 4E), indicating rapid oxidation of the accumulated succinate in both cases. Finally, when the mitochondria-targeted probe MitoB^18,30^ was used to measure H_2_O_2_ production in the heart in vivo, a substantial increase in mitochondrial H_2_O_2_ production upon reperfusion of the ischemic WT hearts was observed, that was not seen upon reperfusion of ND6-P25L hearts (Fig. 4F). Therefore, ND6-P25L mice are protected against cardiac IR injury by the inability of their complex I to catalyze ROS production by RET upon the reperfusion of ischemic tissue.

## Discussion

Here we have described the structural and molecular effects of a single point mutation in a mtDNA-encoded subunit of mammalian complex I. We have determined how its subtle effects create a functionally distinct enzyme that leads to an altered mitochondrial physiology with a major impact on pathophysiology. Therefore, we provide a comprehensive, mechanistic description on every level, directly linking specific molecular changes to their pathophysiological consequences.

On the molecular level, ND6-P25L complex I functions normally for NADH oxidation but is incapable of catalyzing reverse electron transport. It is thus only able to catalyze in one direction, whereas WT complex I not only catalyzes in both directions, but is a thermodynamically reversible catalyst that operates efficiently in either direction in response to only the smallest driving force^31,32^. Importantly, we confirmed that the lack of ROS production by RET in the ND6-P25L strain is directly attributable to complex I itself, because the thermodynamic driving forces and substrates present in ND6-P25L mitochondria are sufficient to drive substantial levels of RET in WT mitochondria. The structure of the ND6-P25L complex indicates how replacing the Pro residue with a Leu pushes ND6 TMHs 2 and 3 apart, allowing the upper section of TMH3 to rotate more easily and facilitating the spontaneous transition into D-like states. In the canonical WT D state, the CoQ-binding site is disordered, and reactivation upon the provision of NADH and CoQ likely includes the substrate acting as a template to reform the site^23^. In contrast, reactivation of the D state under RET conditions (upon the provision of CoQH_2_ and Δp) has not been demonstrated, and occurs either very slowly or not at all. It is therefore possible that ND6-P25L-CI is unable to catalyze RET, even when starting from an A-like state, because (as also may occur during NADH oxidation) it continually visits off-pathway D-like states but (unlike during NADH oxidation) the RET conditions are unable to promptly recover these off-pathway states and return them to catalysis. Alternatively, the ND6-P25L complex may be intrinsically incapable of catalyzing RET (even though it remains fully competent in NADH oxidation). This may be because instability in the conformation of ND6-TMH3 propagates to the ND3-TMH1-2 loop (Cys39 is no longer securely anchored)^24^, compromising its ability to complete the conformational changes that have been proposed to be crucial for catalysis^33^, and/or creating subtle changes at the CoQ-binding site that render it unable to bind CoQH_2_ effectively.

The specific lack of mitochondrial ROS production by RET in the ND6-P25L mouse, caused by a single point mutation that leaves all other aspects of mitochondrial metabolism untouched, makes the ND6-P25L mouse model a powerful resource for investigating its physiological roles and consequences in vivo. Mitochondrial ROS production by RET has been proposed to act as a redox signal in a range of physiological scenarios including inflammation^10,13^, oxygen sensing^11^, thermogenesis^14^ and stress response^15^. In addressing the role of ROS production by RET in vivo, we focused here on the role of complex I in cardiac IR injury^17–19^. RET has been proposed to be central to the mitochondrial ROS production that initiates cardiac IR injury according to the following model: during ischemia the mitochondrial metabolite succinate accumulates, then upon reperfusion it is rapidly oxidized, reducing the CoQ pool and building Δp and thereby driving ROS production by RET through complex I^17,18^. Consistent with this model, the ND6-P25L mutant mouse is protected against cardiac IR injury; it exhibits no change in succinate metabolism, but a substantial decrease in mitochondrial ROS production. Previously, strategies to inhibit succinate accumulation and/or oxidation during IR injury^18,28,34–37^ have also afforded protection against cardiac IR injury, as has inhibition of complex I catalysis^38–40^, albeit by using inhibitors that act in both directions and thereby prevent recovery of normal activity following reperfusion. The ND6-P25L mouse model has thus allowed the precise role of complex I in IR injury to be defined and will now provide crucial opportunities to explore the consequences of RET and mitochondrial ROS production through RET in further physiological settings.

The physiological role and relevance of the deactive transition in complex I has long been debated, especially because much of the evidence for its existence originated from in vitro systems in which extensive incubations are required for the A–D transition^20,21,25,41^. However, we have shown here that a single point mutation greatly enhances the propensity of complex I to enter D-like states in vivo, bypassing the need for extensive incubation, and offering the opportunity to probe their physiological and functional relevance. Importantly, because we have shown that rapidly deactivating complex I is protective against IR injury, destabilizing A-like states relative to D-like states (in analogy to the ND6-P25L mutation) presents a further route for pharmacological intervention against IR injury. It has already been shown that locking WT complex I in the D form by derivatizing Cys39 protects against IR injury^19,41–43^, although this approach still relies on the slow spontaneous deactivation process. In addition, we note that metformin has been reported to preferentially bind the D state of complex I^44^, and is also protective against IR injury in vivo^45,46^, although whether the protection is specifically mediated by complex I remains unclear. Overall, strategies that promote complex I deactivation, to prevent RET but allow reactivation once the conditions for NADH oxidation are restored, are promising therapeutically.

Finally, although this is the first structure of complex I containing a mutation associated with pathology, the single reported human case homoplasmic for this mtDNA variant was subject to a devastating pathology that led to death at eight months. In contrast, the homoplasmic ND6-P25L mouse model used here exhibited only a very mild phenotype with no substantial defect in oxidative phosphorylation, suggesting that epiststatic or environmental effects may have contributed in the single human case. Nevertheless, we have seen that this single amino acid substitution results in a major functional change in the mature complex, highlighting the fact that the propensity of the human enzyme to enter D-like states remains to be determined, and is likely to respond to subtle sequence variations. Thus, human mtDNA polymorphisms and mitochondrial haplotypes affecting this region of complex I could alter individual susceptibilities to RET-related pathologies, such as IR injury, or to inflammatory disorders associated with redox signaling by RET.

In conclusion, by a combination of structural, biochemical and in vivo experiments we have defined the effects of a single point mutation in a ~1MDa respiratory complex that fundamentally alters its molecular and physiological function with major implications for the role of complex I in health and disease.

## Methods

### Mouse strains

The ND6-P25L mouse strain^6^ was generously provided by Professor Douglas Wallace, University of Pennsylvania. ND6-P25L mice were bred and maintained in pathogen-free facilities with a 12 h:12 h light: dark cycle, a room temperature of 19˚C to 22˚C, relative humidity 55% ± 10%, and with *ad lib* food and water. All procedures were approved by the local ethics committees of the MRC Laboratory of Molecular Biology and the University of Cambridge and by the UK Home Office (PPL: P6C97520A). Wild-type (WT) mice (C57BL/6 J) were purchased from Charles River UK, Ltd (Margate, UK). As the mutant mice were backcrossed onto the C57BL/6 J background^6^ both the WT and ND6-P25L mice lack the mitochondrial NAD(P)H transhydrogenase^47^. Mice were used between 8 and 22 weeks and sacrificed by cervical dislocation. All procedures were carried out in accordance with the UK Animals (Scientific Procedures) Act, 1986 and the University of Cambridge Animal Welfare Policy.

### Preparation of mitochondrial membranes from mouse hearts

Hearts were excised from WT or ND6-P25L mice and immersed immediately in ice-cold buffer containing 10 mM Tris-HCl (pH 7.4 at 4 °C), 75 mM sucrose, 225 mM sorbitol, 1 mM EGTA and 0.1% (w/v) fatty acid-free bovine serum albumin (BSA). All the following steps were carried out at 4 °C. Mitochondria were prepared as described previously^48^. The hearts were diced, washed, then homogenized in 10 mL buffer per gram of tissue by seven to ten strokes of a Potter–Elvehjem homogenizer fitted with a Teflon pestle at ~1000 rpm. The homogenate was centrifuged (1000 × *g*, 5 min), then the supernatant was recentrifuged (9000 × *g*, 10 min) to collect the crude mitochondria. The pellets were suspended in resuspension buffer (20 mM Tris-HCl, 1 mM EDTA, 10% glycerol, pH 7.4 at 4 °C) to a protein concentration of ~10 mg mL^−1^ and stored at −80 °C. Mitochondria suspensions were thawed on ice, sonicated using a Q700 Sonicator (Qsonica, US 65% amplitude with three 5-s bursts of sonication interspersed by 30-s intervals on ice) and centrifuged at 75,000 × *g* for 1 h. The pellets containing the mitochondrial membranes were homogenized in resuspension buffer to ca. 5 mg mL^−1^ and stored at −80 °C.

### Preparation of complex I from mitochondrial membranes

ND6-P25L complex I was prepared as described previously^22^. 3–4 mL of membrane suspension were solubilized by addition of 1% dodecyl-β-D-maltoside (DDM, Glycon, Germany), along with 0.005% phenylmethane sulfonyl fluoride (PMSF, Sigma-Aldrich, UK), and centrifuged (32,000 × *g*, 30 min). The supernatant was loaded onto a 1 ml Q-sepharose HP column (GE Healthcare, UK) pre-equilibrated in buffer A (20 mM Tris-HCl (pH 7.14 at 20 °C), 1 mM EDTA, 0.1% DDM, 10% (v/v) ethylene glycol, 0.005% asolectin (Avanti Polar Lipid, USA) and 0.005% CHAPS (Calbiochem, Merck, Germany), and eluted by an increasing proportion of buffer B (buffer A + 1 M NaCl). Complex I eluted in ∼35 % buffer B. The fractions containing complex I were collected, concentrated to 100 μL using a 100 kDa MWCO Vivaspin 500 concentrator (Sartorius, Germany), and eluted from a Superose 6 Increase 5/150GL size exclusion column (GE Healthcare, UK) in 20 mM Tris-HCl (pH 7.14 at 20 °C), 150 mM NaCl and 0.05% DDM. Protein concentrations were determined using the BCA assay (Fisher Scientific UK). The catalytic activity of the isolated enzyme was determined as the rate of NADH:decylubiquinone oxidoreduction, using 0.5 μg mL^−1^ complex I in 20 mM Tris-HCl (pH 7.5 at 32 °C), 0.075% (w/v) asolectin and 0.075% (w/v) CHAPS, and 100 μM decylubiquinone. The reaction was initiated with 100 μM NADH and measured at 340–380 nm (ε = 4.81 mM^−1^ cm^−1^).

### Cryo-EM data collection

UltrAuFoil gold grids (R 0.6/1, Quantifoil Micro Tools GmbH, Germany) were prepared as described previously^23^. Briefly, they were glow discharged (20 mA, 90 s), incubated in a solution of 5 mM 11-mercaptoundecyl hexaethyleneglycol (SensoPath Technologies, USA) in ethanol^49^ for 48 h in an anaerobic glovebox, then washed with ethanol and air-dried just before use. Using an FEI Vitrobot Mark IV, 2.5 μL of ND6-P25L-CI solution (3.5 mg mL^−1^) was applied to each grid at 4 °C in 100% humidity and blotted for 9–10 s at blotting force setting –10, before the grid was frozen by plunging it into liquid ethane. The highest quality cryo-frozen grids were identified using a 200 keV Talos Arctica transmission electron microscope, then high-resolution data acquisition was performed on a 300 keV Titan Krios microscope fitted with a Gatan Quantum K2 Summit detector at the Cambridge University cryo-EM facility. A total of 1519 micrographs were collected for ND6-P25L-CI, each with 40 movie frames, using the FEI EPU software. The calibrated pixel size was 1.054 Å, the defocus range was −1.5 to −3.0 μm and the total electron dose was 50.0 electrons Å^−2^ over a total exposure time of 10 s for each micrograph. Following inspection, 1492 micrographs were retained for analysis.

### Cryo-EM data processing for ND6-P25L

Cryo-EM data processing was carried out using RELION 3.0^50^. 1492 micrographs were motion corrected using Motioncor2 with dose-weighting^50,51^ followed by contrast transfer function (CTF) estimation using Gctf-1.18^52^ with the amplitude contrast set to 8%. 42,622 particles were extracted following template-free automatic particle picking in RELION and manual curation. The particles were 2D classified, and crudely 3D classified with ﻿an angular sampling interval of 3.7° resulting in 37,665 good particles. The particles were re-extracted and a second round of 3D classification using four classes to an angular sampling interval of 0.9° yielded two major classes which were indistinguishable and therefore combined (total of 25,629 particles). The particles were then subject to CTF refinement, Bayesian polishing using all frames, and 3D auto-refinement using EMD-11810 lowpass filtered to 60 Å as an initial model. The final 3D auto-refinement used solvent-flattened FSCs and postprocessing was performed on the final map using a mask made from the deactive mouse model (PDB 7AK5) by the molmap command in UCSF Chimera^53^, and extended by 3 pixels and with an added soft edge of 5 pixels in RELION. The map resolution was 3.82 Å based on the gold standard FSC = 0.143 criterion. The map was further subject to model-free global auto-sharpening using phenix.autosharpen with default parameters to improve map connectivity for model building and refinement.

### Reprocessing of Cryo-EM data for deactive WT complex I

Initial data processing was performed using RELION-3.0 with final steps including CTF refinement performed in version 3.1. 1768 micrographs were motion corrected using the RELION implementation of Motioncorr with and without dose-weighting, followed by CTF estimation on the non-dose-weighted micrographs using Gctf-1.18^52^ with the amplitude contrast set to 8%. 148,440 particles were extracted from dose-weighted micrographs following autopicking in RELION using 46 2D classes from a bovine complex I dataset as a template, followed by manual curation, and 80,603 particles were selected following 2D classification. The particles were then 3D refined using EMD-4356 lowpass filtered to 40 Å as an initial model, and 3D classified crudely to an angular sampling interval of 1.8° to yield 62,595 particles in good classes. The particles were then subject to Bayesian polishing using all frames, and several iterative rounds of refinement and CTF refinement including beam tilt, trefoil and higher order aberration estimations^54^. A second round of 3D classification to an angular sampling interval of 1.8° yielded two classes of similar resolution and number of particles giving a total of 50,184 particles for final refinement with solvent-flattened FSCs. A minor class containing 12,411 particles was refined to a resolution of 3.74 Å, and represents the remaining active state population of the sample. Postprocessing was performed on the final deactive map using a mask made from the updated PDB model by the molmap command in UCSF Chimera^53^, extending by 3 pixels and adding a soft edge of 10 pixels in RELION. The map resolution was 3.17 Å based on the gold standard FSC = 0.143 criterion. To aid model building in Coot, multibody refinement was performed with three bodies to improve density in regions of relatively poor resolution.

### Complex I model building and analyses

Model building and refinement were performed using Coot 0.9-pre^55^ and Phenix-1.16-3549^56^. The active mouse model (PDB 6ZR2) was first crudely fitted into the deactive map using the Chimera fit-in-map command and then rigid-body fitted subunit-by-subunit using Phenix. The model was then subject to cycles of manual adjustment in Coot 0.9-pre using both the globally sharpened map and three multibody maps, and by phenix.realspace.refine with secondary structure restraints against the globally sharpened consensus map. The model, especially the clashscore, was improved by using ISOLDE at an intermediate stage^57^. The final Phenix-refined deactive model was fitted into the ND6-P25L Phenix auto-sharpened map using Chimera fit-in-map and the P25L mutation implemented, then it was subjected to cycles of manual and automated refinement as above. Model-to-map FSC curves were generated by creating a map at Nyquist frequency from the model using molmap in UCSF Chimera. The created map was compared to an unfiltered, unsharpened, masked experimental consensus map from RELION by using the Xmipp tool in SCIPION 1.2^58^. Final model statistics were produced by Phenix-1.16-3549, MolProbity 4.4^59^ and EMRinger^60^. The structures were analyzed using Coot, UCSF Chimera and PyMol^61^ and movies created in UCSF Chimera X^62^.

### Catalytic activity measurements on mitochondrial membranes

All assays were carried out in 10 mM Tris-SO_4_ (pH 7.4) and 250 mM sucrose at 32 °C in a SPECTRAmax PLUS384 spectrophotometer (Molecular Devices, UK). NADH:O_2_, succinate:O_2_ and NADH:APAD^+^ (3-acetylpyridineadenine dinucleotide) oxidoreduction were determined using 10 μg-protein mL^−1^ membranes supplemented by 1.5 μM horse heart cytochrome *c* and 15 μg mL^−1^ alamethicin, unless otherwise specified. The rate of oxidation of 200 μM NADH was monitored at 340–380 nm (ε = 4.81 mM^−1^ cm^−1^) and confirmed to be fully inhibitor sensitive by addition of 1 μM piericidin A. The rate of oxidation of 5 mM succinate was determined using a coupled assay system comprising 90 μg mL^−1^ fumarate hydratase (FumC) and 500 μg mL^−1^ oxaloacetate decarboxylating malic dehydrogenase (MaeB) with 2 mM MgSO_4_ and 2 mM K_2_SO_4_^63^, by monitoring the coupled reduction of 2 mM NADP^+^ at 340–380 nm (ε = 4.81 mM^−1^ cm^−1^) and confirmed to be fully inhibitor sensitive by addition of 2 μM atpenin A5 (Santa Cruz Biotechnology, USA). NADH:APAD^+^ oxidoreduction was monitored using 500 μM APAD^+^ and 200 μM NADH in the presence of 1 μM piericidin A at 400–450 nm (ε = 3.16 mM^−1^ cm^−1^). The rate of NADH-driven H_2_O_2_ production was monitored by the horseradish peroxidase (HRP)-dependent oxidation of Amplex Red to resorufin at 557–620 nm (ε = 51.60 mM^−1^ cm^−1^)^64^, using 40 μg-protein mL^−1^ membranes with 2 U mL^−1^ HRP, 10 μM Amplex Red, 10 U mL^−1^ superoxide dismutase from bovine erythrocytes (SOD), 0.5 μM piericidin A and 30 μM NADH. Background rates measured in the presence of catalase and the absence of membranes were subtracted.

Complex I was deactivated in membranes^23^ by incubating the membrane suspension (5 mg-protein mL^−1^) for 30 min at 37 °C in the absence of substrates. Complex I was pre-activated in membranes by diluting the membranes to 2 mg-protein mL^−1^ in assay buffer, adding 1 mM NADH and incubating at room temperature for 10 sec. To determine the A/D ratio, the membranes were diluted to 2 mg-protein mL^−1^ if necessary, then 0.5 μL of 200 mM *N*-ethylmaleimide (NEM) in DMSO were added to one 50 μL aliquot, and 0.5 μL of DMSO added to a second control aliquot. The two aliquots were incubated on ice for 20 min and their rates of NADH oxidation determined as above. The A/D ratio was calculated by assuming that in the NEM-treated sample only the complex in the A-state is capable of turnover, whereas in the control sample both the A- and D-states are capable.

### Isolation of intact mitochondria from heart tissue

Mitochondria for functional analysis were isolated, with all steps performed on ice, by homogenization of freshly retrieved mouse heart tissue (12–20-week-old male wild-type C57BL/6 J or ND6-P25L mice) using an all-glass dounce homogenizer in STE buffer (250 mM sucrose, 5 mM Tris-Cl, 1 mM EGTA, pH 7.4 at 4 °C) supplemented with 0.1% (w/v) fatty acid-free BSA (STEB buffer). Homogenates were pelleted twice by centrifugation at 700 × *g* for 5 min (4 °C), then the supernatant collected and centrifuged at 5500 × *g* for 10 min (4 °C). The mitochondrial pellets were then resuspended in 1 mL STEB buffer and re-centrifuged at 5500 × *g* for 10 min (4 °C) prior to their final re-suspension in 80 μL STE buffer per heart. Protein concentrations were measured using the bicinchoninic acid (BCA) assay with bovine serum albumin (BSA) as a standard.

### Combined mitochondrial O_2_ consumption and ROS measurements

For combined measurement of O_2_ consumption and ROS production a high resolution O2K Oxygraph with attached fluorescence LED module (Oroboros Instruments, Austria) was used. Mitochondria (0.15 mg-protein/mL) were incubated in 2 mL KCl buffer (120 mM KCl, 10 mM HEPES, 1 mM EGTA, pH 7.2 at 37 °C) supplemented with 40 μg/mL superoxide dismutase (Cu, Zn SOD), 20 μg/mL horseradish peroxidase (HRP), 200 μg/mL fatty acid free BSA, 12.5 μM Amplex Red while stirring at 37 °C. Chambers were closed and respiration was started by addition of 0.5 mM glutamate and malate, followed by addition of 10 mM succinate after 5 min. The O_2_ concentration was calibrated assuming equilibration with air in the open chamber and taking zero oxygen as the point when all oxygen was depleted by mitochondria in the closed chamber. Resorufin fluorescence was measured via the O2K fluorometer 525 nm LED modules and signal was calibrated via titration of known amounts of H_2_O_2_ (0–3 μM) in the presence of mitochondria, fatty-acid free BSA, HRP and Amplex Red.

### Ischemic heart incubations

Hearts were retrieved from 8- to 16-week-old male wild-type C57BL/6 J or ND6-P25L mice following cervical dislocation. For labeling of exposed cysteine residues, the hearts were cut longitudinally into five equal slices, and the 0 min slice clamp frozen straight away at liquid nitrogen temperature. The residual pieces were incubated in the chest of the warmed (37 °C) mouse on a temperature-controlled heat pad for up to 30 min, then frozen. For the analysis of CoQ and succinate in ischemic hearts, the hearts were cut in half with one-half clamp frozen as the normoxic sample while the other half was placed back in the chest of the animal for 30 min as above and subsequently analyzed for succinate levels and CoQ redox state.

### Labeling of exposed cysteine residues

For labeling of exposed cysteine residues, frozen heart tissue (~5 mg) was homogenized in 400 μl of ice cold 50 mM KPi buffer (pH 7.8 at 30 °C) containing 20 mM light iodoacetamide (Sigma-Aldrich, UK) and 10 mM TCEP, using a Precellys24 tissue homogenizer (6500 rpm, 15 s; Bertin Instruments, France) and lysis tubes (CK-14, Bertin Instruments, France). Cysteines were labeled for 5 min on ice then the reaction was quenched by addition of 1 mL of KPi buffer. The membranous fraction was pelleted at 17,000 × *g*, 4 °C for 5 min, the pellets washed with 1 mL of KPi buffer, and centrifuged as before. All residual thiols were labeled by resuspending the pellets in 45 μL of lysis buffer (4% SDS, 50 mM NaPi, pH 7.8 at 37 °C) containing 20 mM heavy (^13^C_2_, 2-d_2_) iodoacetamide (Sigma-Aldrich, UK) and 10 mM TCEP and incubated at 37 °C for 30 min. Following addition of the appropriate loading dye, samples were subjected to SDS-PAGE, then fixed in 50% methanol and 10% acetic acid and stained with QC Colloidal Coomassie Stain (BioRad, UK). Gel sections between the 10 and 20 kDa marker bands (Precision Plus Protein™ Dual Color Standard, BioRad, UK) were excised and the proteins were in-gel digested with trypsin. The extracted peptides were desalted with C18 tips (OMIX C18, Agilent, UK) according to the manufacturer’s instructions and dissolved in MS sample buffer (20% acetonitrile, 0.1% formic acid) followed by MS analysis. Light and heavy iodoacetamide-labeled tryptic ND3 peptides were analyzed using a Xevo TQ-S triple-quadrupole mass spectrometer with the attached I-Class ACQUITY UPLC^®^ system (both Waters, UK). The peptides were separated by reverse-phase at 30 ˚C on an ACQUITY UPLC^®^ BEH C18 column (1.7 µM, 130 Å, 1 × 50 mm; Waters, UK). MS buffer A (5% acetonitrile, 0.1% formic acid) and MS buffer B (90% acetonitrile, 0.1% formic acid) were used at 200 μL/min for the following LC gradient: 0–0.3 min, 5% buffer B; 0.3–3 min, 5%-100% buffer B; 3–4 min, 100% buffer B; 4.0–4.10 min, 100%–5% buffer B; 4.10–4.60 min, 5% buffer B. Eluant was diverted to waste at 0–1 min and 4–5 min. The peptides were detected by multiple reaction monitoring in positive ion mode using the following settings: source spray voltage 3.0 kV; cone voltage 2 V; ion source temperature 150 ^◦^C; collision energy 25 V. For quantification the following transitions were used: light labeled ND3 peptide, 836.7 > 744.0; heavy labeled ND3 peptide, 838.7 > 746.0. The peak areas of light and heavy labeled ND3 peptides were quantified using the MassLynx 4.1 software (Waters, UK) and the proportion of light-labeled peptides determined.

### Mitochondrial ROS measurements

Measurements of H_2_O_2_ production by mitochondria were performed at room temperature using a fluorometric plate reader to assess the conversion of Amplex Red to resorufin (CLARIOstar, BMG Labtech, Germany). Heart mitochondria (30 μg protein) were incubated with 4 μg HRP, 8 μg superoxide dismutase (Cu,Zn-SOD) and 40 μg fatty acid free BSA in a final volume of 100 μL KCl buffer in a 96-well plate. A further 100 μL KCl buffer was added containing 10 mM succinate or 10 mM (each, or 0.5 mM if specified) glutamate/malate, 12.5 μM Amplex Red, and inhibitors (5 µM of FCCP or rotenone) as indicated. Alternatively, 10 mM succinate was added following priming with glutamate/malate. Resorufin fluorescence (excitation at 560 nm, emission at 590 nm) was calibrated against known H_2_O_2_ concentrations (ε_240_ = 43.6 M^−1^ cm^−1^).

### CoQ extraction from isolated mitochondria

CoQ extraction was performed as described previously^26^. Mitochondria (15 μg protein) were incubated for 5 min at 37 °C in 100 μL KCl buffer (120 mM KCl, 10 mM HEPES, 1 mM EGTA, pH 7.2 at 37 °C), with succinate (10 mM) and rotenone (1 μM) if indicated. Incubations were transferred to ice-cold extraction solution (200 μL of acidified methanol and 200 μL hexane) and vortexed. The hexane phase was separated by centrifugation (5 min, 17,000 × *g*, 4 °C), collected, dried down in 1 mL glass mass spectrometry vials (186005663CV, Waters, UK) under a stream of nitrogen and the CoQ extract was resuspended in methanol containing 2 mM ammonium formate followed by LC-MS analysis.

### LC-MS/MS analysis of CoQ redox state

CoQ redox state determination by LC-MS was performed as described previously^26^. LC-MS/MS analyses were carried out using an I-Class ACQUITY UPLC^®^ system attached to a Xevo TQ-S triple quadrupole mass spectrometer (both Waters, UK). Samples were kept at 8 °C prior to injection by the autosampler of 2–10 μL into a 15 μL flow-through needle and separated by reverse-phase at 45 °C using an ACQUITY UPLC^®^ BEH C18 column (1.7 μM, 130 Å, 2.1 × 50 mm; Waters, UK). Mobile phase was isocratic 2 mM ammonium formate in methanol run at 0.8 mL/min over 5 min. For MS analysis, electrospray ionization in positive ion mode was used with the following settings: capillary voltage 1.7 kV; cone voltage 30 V; ion source temperature 100 °C; collision energy 22 V. Multiple reaction monitoring in positive ion mode was used for compound detection. Transitions used for quantification were: CoQ_9_, 812.9 > 197.2; CoQ_9_H_2_, 814.9 > 197.2. Samples were quantified using MassLynx 4.1 software (Waters, UK) to determine the peak areas for CoQ_9_ and CoQ_9_H_2_.

### Membrane potential measurement in isolated mitochondria

Membrane potentials in isolated mitochondria were measured by the accumulation of radiolabeled triphenylmethyl phosphonium ([^3^H]-TPMP, American Radiolabeled Chemicals, USA) as described previously^65,66^. Mitochondria (100 μg) were incubated for 5 min at 37 °C in KCl buffer (120 mM KCl, 10 mM HEPES, 1 mM EGTA, pH 7.2 at 37 °C), with succinate (10 mM), 5 μM TPMP (cold), 50 nCi/mL [^3^H]-TPMP and, if indicated, 0.5 μM rotenone. After incubations mitochondria were pelleted by centrifugation (10,000 × *g* for 1 min, RT) and 200 μL of the supernatant was collected. Residual liquid was carefully removed and pellets were lysed in 40 μL of 20% Triton X-100 followed by addition of 160 μL H_2_O. Scintillation of pellet and supernatant was measured in a Tri-Carb 2800 R liquid scintillation analyzer (PerkinElmer, US) after addition of 3 mL scintillant. The membrane potential was calculated based on the measured scintillation counts using the following Eq. 1 and assuming a mitochondrial volume of 0.6 μL/mg protein and a TPMP binding correction of 2.1^66^:

1 $$\Delta {\Psi}\left( {{\mathrm{mV}}} \right) = 61.5 \times {\mathrm{log}}_{10}\left( {\frac{1}{{2.1}} \times \frac{{\left[ {\left[ {{\,}^{3}{\mathrm{H}}} \right]{\mathrm{TPMP}}} \right]_{{\mathrm{in}}}}}{{\left[ {\left[ {{\,}^{3}{\mathrm{H}}} \right]{\mathrm{TPMP}}} \right]_{{\mathrm{out}}}}}} \right) = 61.5 \times {\mathrm{log}}_{10}\left( {\frac{1}{{2.1}} \times \frac{{\left( {\frac{{\left[ {{\,}^{3}{\mathrm{H}}} \right]{\mathrm{TPMPcounts}}_{{\mathrm{in}}}}}{{{\mathrm{Mito}}{\mathrm{.}}\,{\mathrm{vol}}{\mathrm{.}}}}} \right)}}{{\left( {\frac{{\left[ {{\,}^{3}{\mathrm{H}}} \right]{\mathrm{TPMPcounts}}_{{\mathrm{out}}}}}{{{\mathrm{Buffer}}\,{\mathrm{vol}}{\mathrm{.}}}}} \right)}}} \right)$$

The calculated membrane potentials were lower than reported previously for rat heart mitochondria^8^, which we ascribe to less stringent purification due to low the yields obtained from mouse heart leading to a greater background protein contamination.

### LAD open-chest mouse model of acute myocardial IR injury

All procedures were carried out in accordance with the UK Home Office Guide on the Operation of Animal (Scientific Procedures) Act 1986 and have been approved by the University of Cambridge Animal Welfare Policy under license number 70/8238. Age and sex matched C57BL/6 J mice ranging between 9 and 20 weeks (22–32 g) were obtained from Charles River. An open chest model of acute myocardial ischemia/reperfusion injury was modified and used as described previously^67^. In short, mice were anesthetized with sodium pentobarbital (70 mg/kg intraperitoneal); following endotracheally intubation, ventilation at 110 breaths per minute (tidal volume 125–150 µL, dependent on weight), a sternal thoracotomy was performed, and the major branch of the left anterior descending (LAD) coronary artery was occluded for 30 minutes followed by 2 h of reperfusion as previously described^66^. Areas at risk were identified by infusion of Evans Blue following LAD re-ligation and infarct sizes were determined in a blinded fashion by triphenyltetrazolium chloride staining and expressed as a proportion of the area at risk^29,67^. For mitochondrial H_2_O_2_ analyses, 50 nmol MitoB was injected I.V. via the tail vein prior to the hearts being exposed to either 30 min ischemia or ischemia followed by 15 min reperfusion. Both conditions allowed MitoB to be present for 55 min prior to the end of the experiment^18^. Hearts were snap-frozen in liquid nitrogen and mitochondrial ROS was assessed by determination of the MitoP/MitoB ratio by LC-MS/MS relative to deuterated internal standards.

### Succinate quantification

Succinate was extracted from tissues samples and quantified as described previously^68^. Briefly, tissues were homogenized in MS extraction buffer (50% methanol, 30% acetonitrile and 20% H_2_O), supplemented with an internal standard of 1 nmol of [^13^C_4_]-succinate (Sigma Aldrich, UK); 25 μL/mg wet weight tissue) in a Precellys 24 tissue homogenizer (6500 rpm, 15 s; Bertin Instruments, France). The homogenate was agitated on a shaking heat block (1400 rpm, 4 °C, 15 min) and then incubated (−20 °C, 1 h). The sample was then centrifuged (17,000 × g, 10 min, 4 °C), the pellet discarded and the centrifugation repeated. The resulting supernatant was transferred to pre-cooled MS vials and stored at –80 °C until analysis. Succinate analysis was carried out using a LCMS-8060 mass spectrometer with a Nexera X2 UPLC system (both Shimadzu, UK). 5 μL of sample were injected onto a SeQuant^®^ ZIC^®^HILIC column (3.5 μm, 100 Å, 150 × 2.1 mm, 30 °C column temperature) with a ZIC^®^-HILIC guard column (200 Å, 1 × 5 mm; both MerckMillipore, UK). Separation was achieved with mobile phases of A) 10 mM ammonium bicarbonate and B) 100% acetonitrile running at a gradient of 0–0.1 min, 80% B; 4 min, 20% B; 10 min, 20% B; 11 min, 80% B; 15 min, 80% B. The mass spectrometer was operated in negative ion mode with multiple reaction monitoring and data acquired and analyzed using Labsolutions software (Shimadzu, UK). Succinate was quantified using standard curves with known amounts of succinate compared against the internal standard.

### CoQ extraction from tissue

CoQ extraction and redox state analysis were performed as described previously^26^. Clamp frozen heart tissue (in situ ischemic heart model; ~5 mg) was weighed into cooled lysis tubes (CK-14, Bertin Instruments, France) on dry ice. Then a mixture of 250 μL ice-cold acidified methanol and 250 μL hexane was added, and tissue was rapidly homogenized in a Precellys24 tissue homogenizer (6500 rpm, 15 s; Bertin Instruments, France). The upper, CoQ-containing hexane layer was separated by centrifugation (5 min, 17,000 × *g*, 4 °C), dried down in 1 mL glass mass spectrometry vials (186005663CV, Waters, UK) under a stream of nitrogen and the CoQ extract was resuspended in methanol containing 2 mM ammonium formate for LC-MS analysis.

### Langendorff-perfused mouse hearts

Langendorff-perfusion of mouse hearts was carried out as described previously^68^. All procedures were carried out with the approval of the Queen Mary, University of London local ethics committee and the UK Home office, under licence PPL PB137135C. Mice were administered terminal anesthesia via intra-peritoneal pentobarbitone injection (~140 mg/kg body weight). Beating hearts were rapidly excised, cannulated and perfused in isovolumic Langendorff mode at 80 mm Hg pressure maintained by a STH peristaltic pump controller feedback system (AD Instruments, UK), with phosphate-free Krebs–Henseleit (KH) buffer continuously gassed with 95% O_2_/5% CO_2_ (pH 7.4, 37 °C) containing (in mM): NaCl (116), KCl (4.7), MgSO_4_.7H_2_O (1.2), NaHCO_3_ (25), CaCl_2_ (1.4), glucose (11). Cardiac function was assessed using a fluid-filled cling-film balloon inserted into left ventricle (LV) connected via a line to a pressure transducer and a Powerlab system (AD Instruments, UK). The volume of the intraventricular balloon was adjusted using a 1.0 mL syringe to achieve an initial LV diastolic pressure (LVDP) of 4–9 mm Hg. Ex vivo cardiac functional parameters (systolic pressure, end diastolic pressure, heart rate, coronary flow, perfusion pressure) were monitored throughout the experiment using LabChart software v.7 (AD Instruments, UK) and analysed using Microsoft Excel software 1.16.16.15. After 20 min equilibration (normoxia), hearts were either subjected to 20 min global no-flow ischemia only, or 20 min ischemia followed by 6 min reperfusion. Hearts were subsequently snap frozen using Wollenberger tongs, pre-cooled in liquid nitrogen and stored at −80 °C until further analysis.

### Statistical analysis

Statistical comparison between two groups was carried out using GraphPad Prism 8 software (GraphPad Software, USA) using two-tailed Student’s *t*-tests. Multiple groups were compared using a two-way ANOVA with Tukey’s multiple comparisons test. The number of biological replicates (*n*) and statistical values and tests are defined in each figure legend.

### Reporting summary

Further information on experimental design is available in the Nature Research Reporting Summary linked to this paper.

## Supplementary information

Supplementary Information

Description of Additional Supplementary Files

Supplementary Movie 1

Reporting Summary

## Data Availability

The structure coordinates and Cryo-EM maps generated during this study are available at the RCSB PDB with accession codes: EMD-11811, PDB ID: 7AK6 (ND6-P25L) and EMD-11810, PDB ID: 7AK5 (deactive state). Associated raw cryo-EM micrograph images are available as EMPIAR entries EMPIAR-10605 (ND6-P25L) and EMPIAR-10604 (Deactive). Source data are provided with this paper.

## Source data

Source Data
