# Supplementary material for: Structural basis for a complex I mutation that blocks pathological ROS production
Source: Nat Commun. 2021 Jan 29;12:707. doi: 10.1038/s41467-021-20942-w (PMC7846746; doi:10.1038/s41467-021-20942-w)
Supplement: Supplementary file 1 — Supplementary Information [file 41467_2021_20942_MOESM1_ESM.pdf]

Supplementary Information

Structural basis for a complex I mutation that blocks pathological ROS production

Zhan Yin[1], Nils Burger[1], Duvaraka Kula-Alwar[2], Dunja Aksentijević[3,4], Hannah R. Bridges[1], Hiran A. Prag[1], Daniel N. Grba[1], Carlo Viscomi[1], Andrew M. James[1], Amin Mottahedin[1,2,5], Thomas Krieg[2], Michael P. Murphy[1,2*] & Judy Hirst[1*]

[1]MRC Mitochondrial Biology Unit, University of Cambridge, Cambridge Biomedical Campus, CB2 0XY, UK

[2]Department of Medicine, University of Cambridge, Cambridge, CB2 0QQ, UK

[3]William Harvey Research Institute, Barts and The London School of Medicine and Dentistry, Queen Mary University of London, Charterhouse Square, London, EC1M 6BQ, UK

[4]Centre for Inflammation and Therapeutic Innovation, Queen Mary University of London, Charterhouse Square, London, EC1M 6BQ, UK.

[5]Department of Physiology, Institute of Neuroscience and Physiology, Sahlgrenska Academy, University of Gothenburg, Gothenburg, Sweden

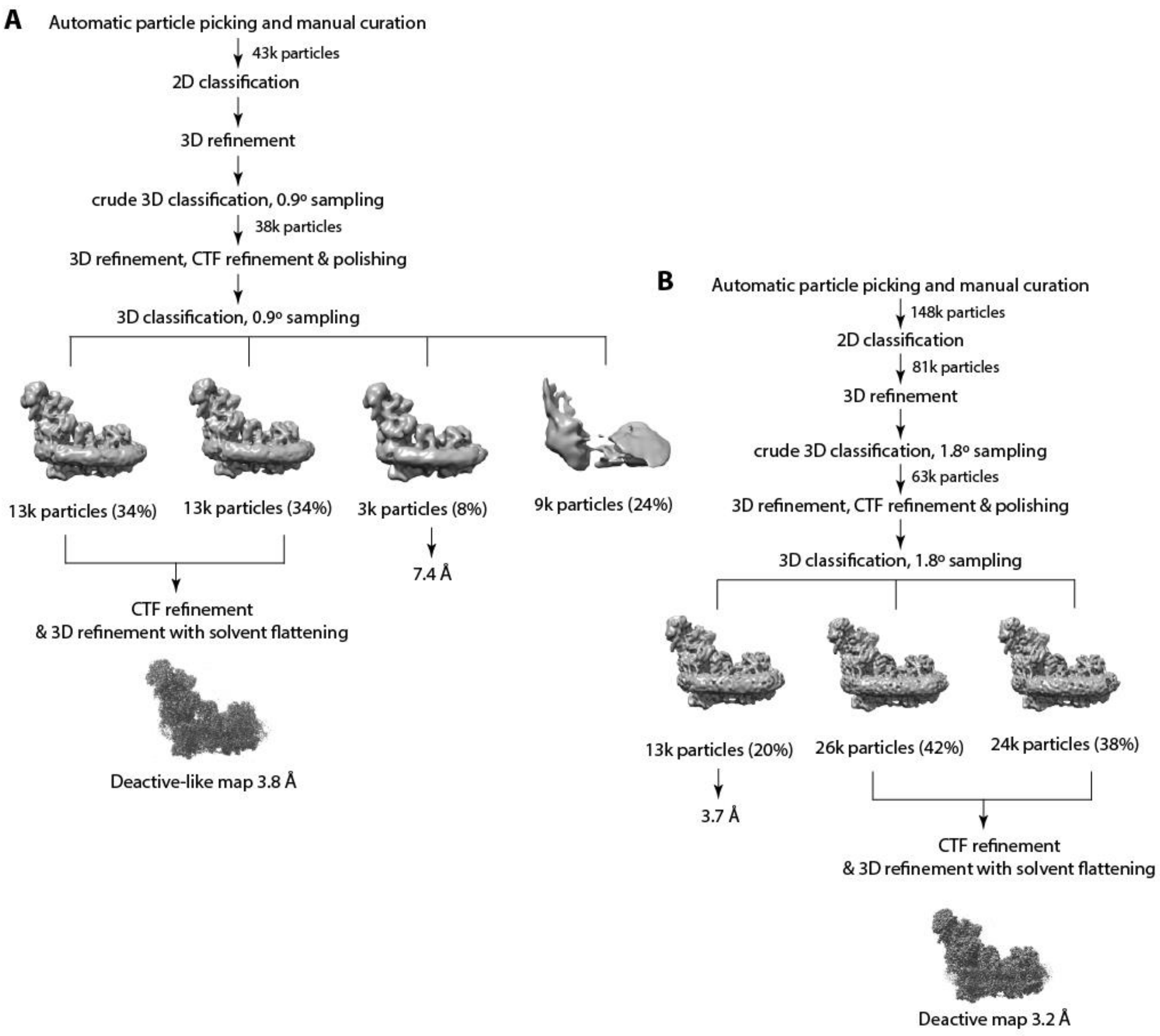

Supplementary Fig. 1. Classification and Refinement Scheme for Cryo-EM Data Processing for ND6-P25L-CI and the deactive State of WT-CI.

(A) ND6-P25L-CI. (B) Deactive state of WT-CI. Based on the relative positions of subunit NDUFA10 and NDUFA5 the 7.4 Å resolution map from a minor class of ND6-P25L-CI particles is also in a deactive-like state, and the 3.7 Å resolution map from a minor class in the deactive preparation of WT-CI most likely results from a mixed class of active- and deactive-like particles. Related to Figure 1.

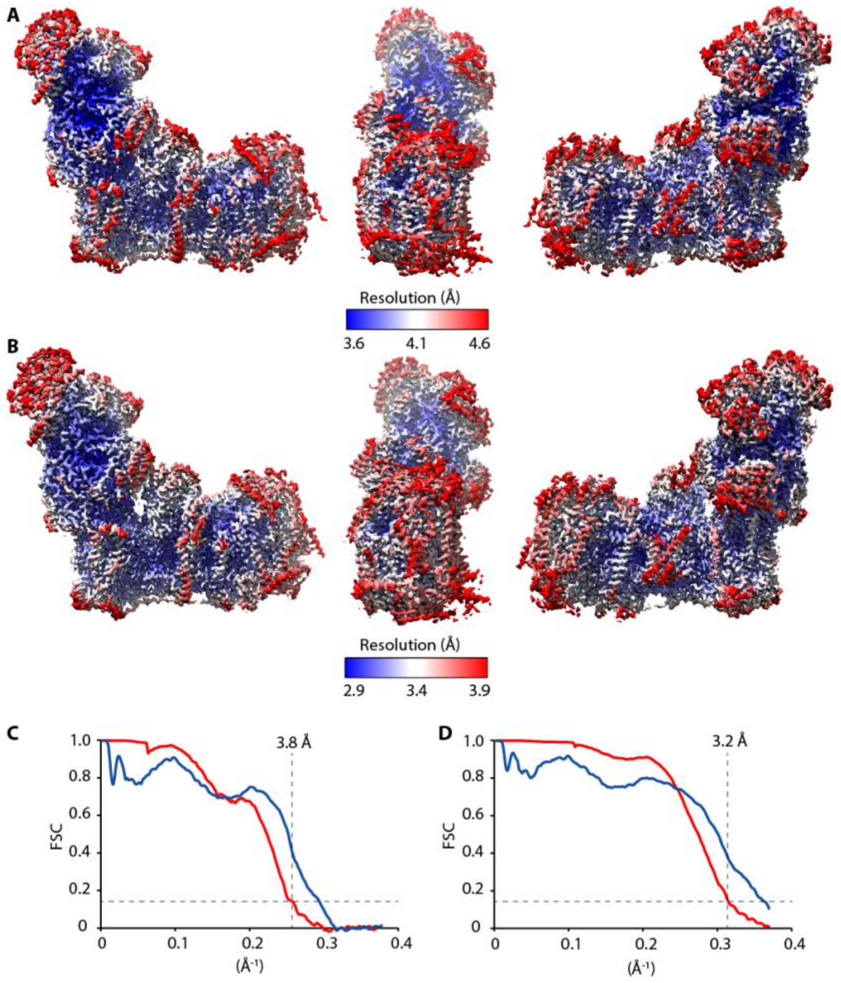

Supplementary Fig. 2 Resolution Estimates of the Cryo-EM Density Maps for the ND6-P25L-CI and the deactive state of WT-CI.

(A, C) ND6-P25L-CI. (B, D) Deactive state of WT-CI. In (A) and (B), local resolutions were estimated using the Local Resolution function in RELION with default parameters and shown using UCSF Chimera according to the color bar. In (C) and (D) the estimated global map resolutions, defined where the red FSC line = 0.143, are 3.8 and 3.2 Å, respectively. In both cases, the refined models agree well with the maps, as shown by the map vs. model FSC curves (blue). Related to Figure 1.

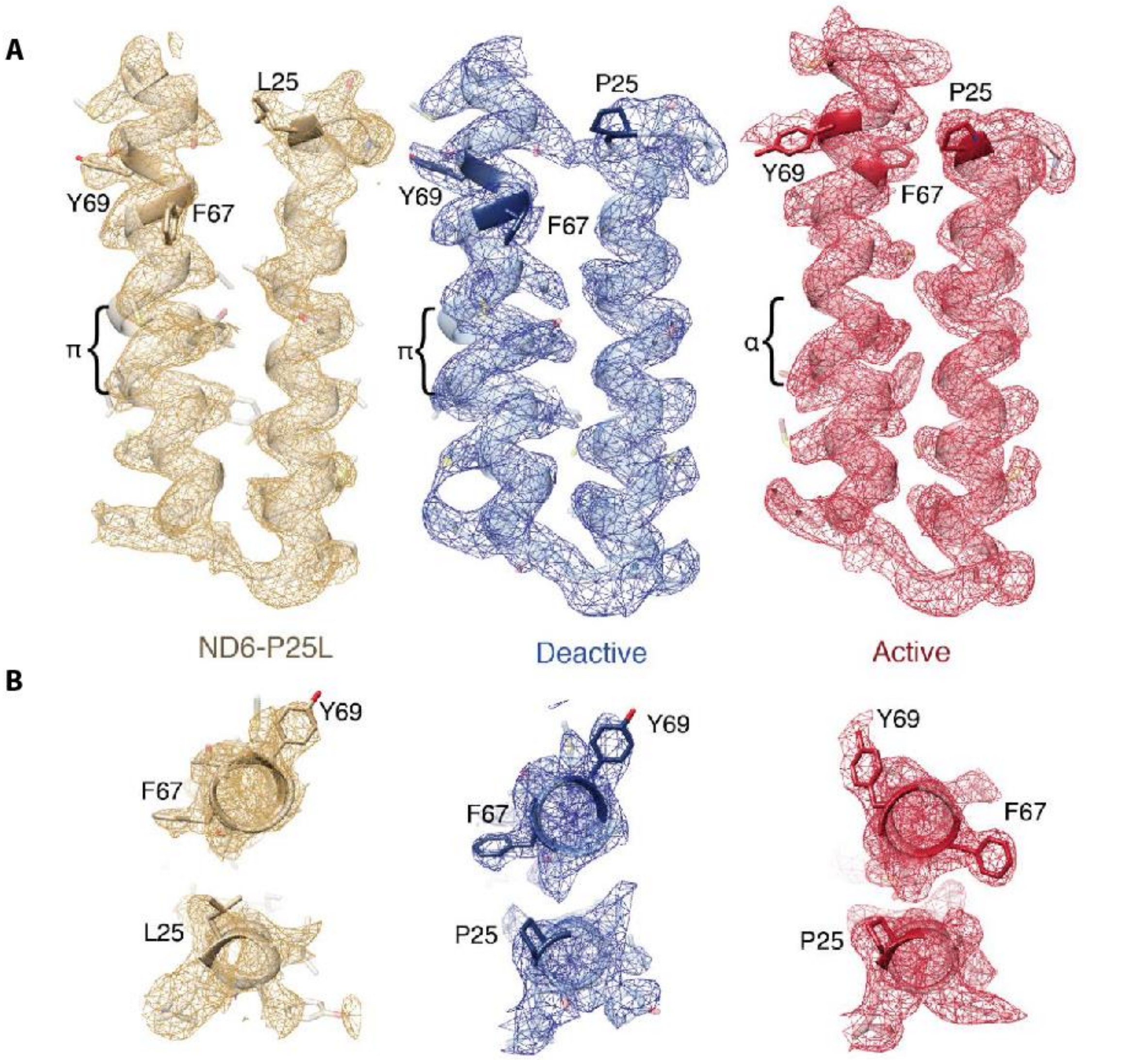

Supplementary Fig 3. Models and densities for ND6 TMHs 2 and 3 showing the relative positions of bulky residues and the mutation site.

(A) side view from the membrane plane; (B) top view from the matrix side. Densities were rendered 2 Å from the model in UCFS Chimera at thresholds of 0.0162, 0.0238, and 0.0161 for ND6-P25L, deactive and active, respectively. The mesh was surfaced smoothed with two iterations with a factor of 0.3. Residues are shown as transparent except for P/L25, F67 and Y69. Related to Figure 1.

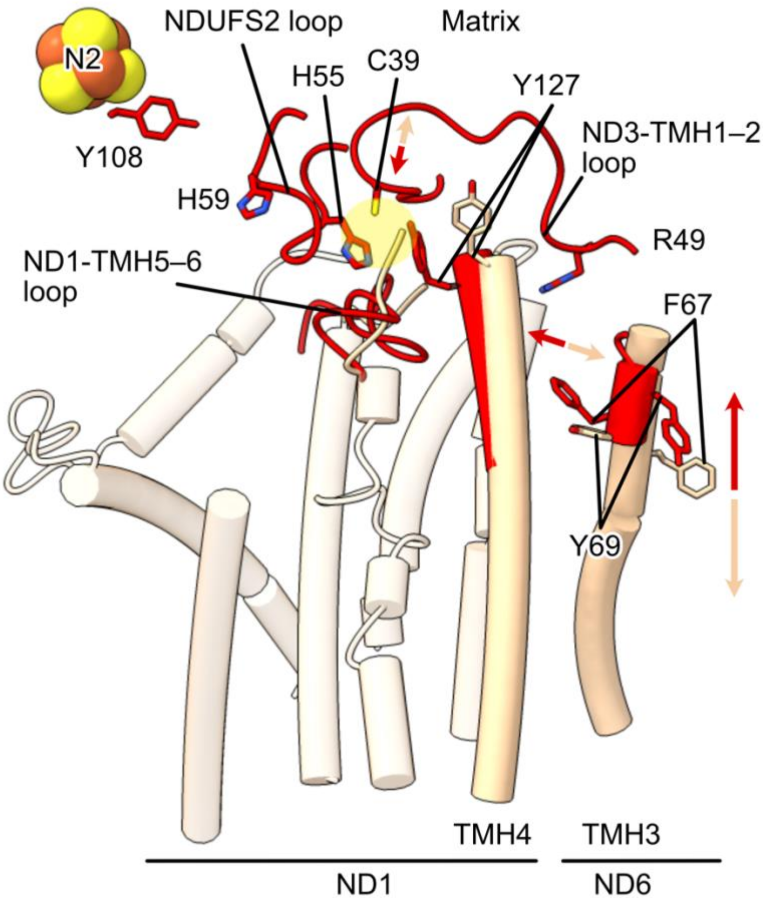

Supplementary Fig. 4. Structural adjustments associated with rotation of the ND6-TMH3 π bulge. The rotation of the ND6-TMH3 π bulge in the ND6-P25L complex I (wheat) (and also in the deactive wild-type enzyme, not shown) when it adopts an α-helical structure in the active state (red) results in an upward motion and reorientation of bulky residues F67 and Y69. There is an accompanying shift in the upper portion of ND1-TMH4, resulting in Y127 forming a binding pocket (yellow highlight) for the Cys39 on the ND3-TMH1–2 loop, along with NDUFS2-H55. This binding site is formed when ordered ND1-TMH5–6, NDUFS2 and ND3-TMH2–3 loops are present in the active site. All of these loops are disordered and exposed to the matrix in the ND6-P25L enzyme and in the deactive state (not shown), resulting in destabilization of the Cys39-binding pocket (note the partial ND1-TMH5–6 loop of the ND6-P25L enzyme in the destroyed binding pocket), straightening of ND1-TMH4 and formation of the ND6–TMH3 π bulge. The ubiquinone catalytic ligands (Y108 and H59) and the terminal N2 cluster are shown for reference. Colored arrows indicate the general motion observed in transitions to the active state (red) and the P25L/deactive states (wheat). Related to Figure 1.

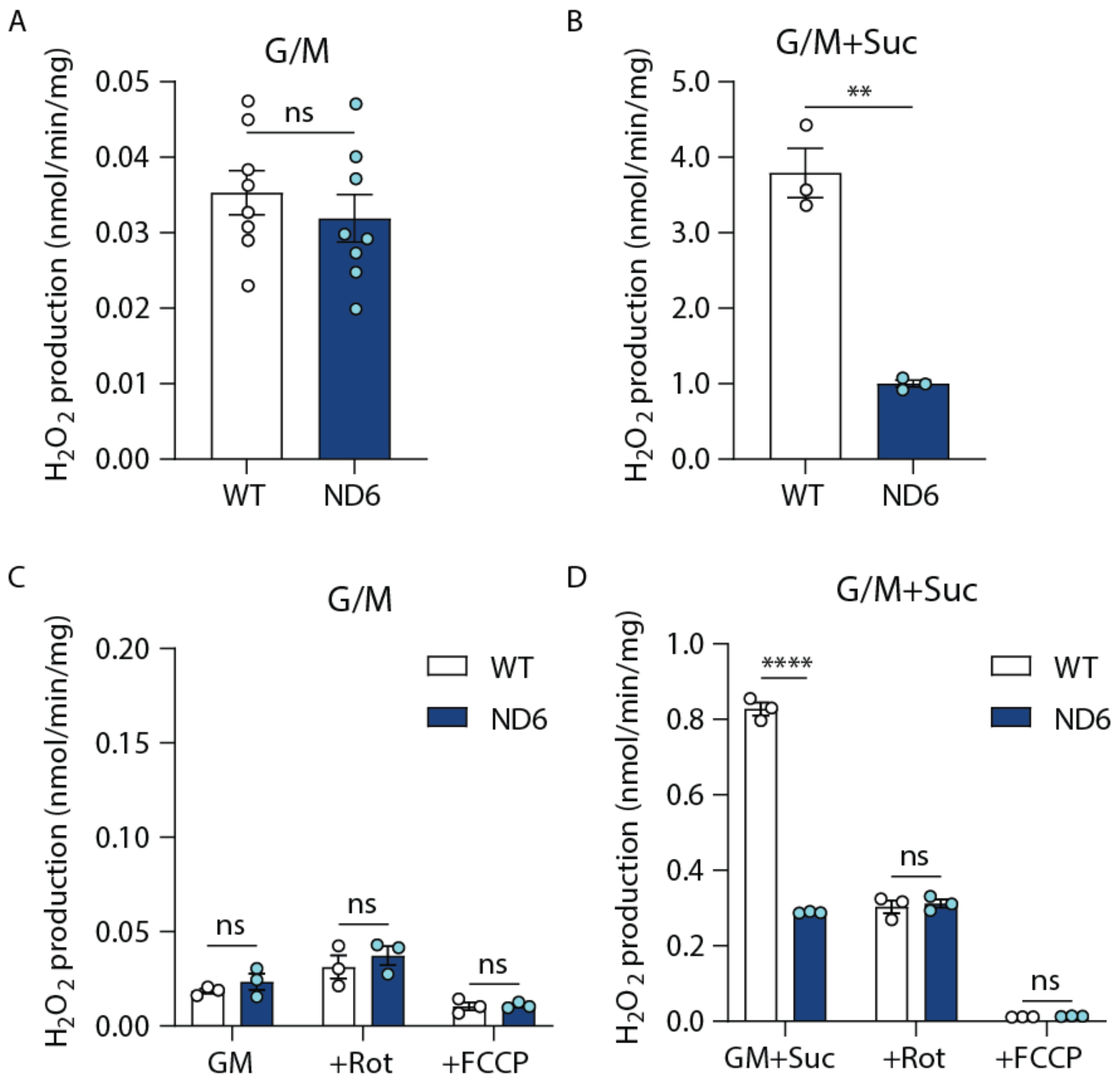

Supplementary Fig. 5 Quantification of Mitochondrial H_2O_2 Production.

(A, B) The rates of H_2O_2 production by isolated heart mitochondria at 37 °C in the O2K Oxygraph, during respiration on (A) glutamate/malate (0.5 mM) and (B) upon subsequent addition of succinate (10 mM). Mitochondria were isolated from four hearts and pooled to provide a single test sample. The data are mean averages ± S.E.M. (A, n = 8, B, n = 3, technical replicates) evaluated using an unpaired, two-tailed Student's t-test (**, $p < 0.01$). In A, $p = 0.439$ and in B, $p = 0.001$. One of the replicates is shown in Figure 3F. (C, D) The rates of H_2O_2 production by isolated heart mitochondria at room temperature in the Amplex Red plate reader assay, during respiration on (C) glutamate/malate (0.5 mM) and (D) upon subsequent addition of succinate (10 mM). Rotenone or FCCP (at 5 μM) were added as indicated. The data are mean averages ± S.E.M. (n = 3, where each replicate is from an independent mitochondrial preparation on a different heart) evaluated using a two-way ANOVA test with Tukey's multiple comparisons correction (****, $p < 0.0001$). The p values in C are: 0.939, 0.875 and 0.999, respectively. The p values in D are: <0.0001, 0.993 and 0.999, respectively. Related to Figure 3.

Supplementary Table 1

Cryo-EM Data Collection, Refinement and Validation Statistics. Related to Figure 1.

	**WT Deactive**<br>EMD-11810<br>PDB 7AK5	**ND6-P25L**<br>EMD-11811<br>PDB 7AK6
**Data collection and processing**		
Magnification	37,600	130,000
Voltage (kV)	300	300
Electron exposure (e–/Å^2)	50	50
Defocus range (μm)	-2.2 to -3.4	-1.5 to -3.0
Pixel size (Å)	1.350	1.054
Symmetry imposed	*C*1	*C*1
Initial particle images (no.)	148,440	42,622
Final particle images (no.)	50,184	26,638
Map resolution (Å)	3.18	3.83
FSC threshold	0.143	0.143
Map resolution range (Å)	2.9 to 7.6	3.5 to 8.2
Map sharpening *B* factor (Å^2)	-23	-74
**Refinement**		
Initial model used	PDB 6ZR2	PDB 6ZR2
Model resolution (Å)	3.2	3.9
FSC threshold	0.5	0.5
Model composition		
Nonhydrogen atoms	65,903	65,341
Protein residues	8,070	8,063
Ligands	28	21
B factors mean (Å^2)		
Protein	42.79	58.21
Ligand	38.24	57.59
R.m.s. deviations		
Bond lengths (Å)	0.003	0.004
Bond angles (°)	0.587	0.605
Validation		
MolProbity score	1.80	2.1
Clashscore, all atoms	7.25	10.83
Poor rotamers (%)	0.01	0
Ramachandran plot		
Favored (%)	94.05	89.91
Allowed (%)	5.94	10.07
Disallowed (%)	0.01	0.01
EMRinger	2.66	2.19

Supplementary Table 2

Summary of the models for the subunits of deactive and ND6 mouse complex I. Where the values differ they are given first for the deactive model, then in brackets for the ND6 model. Related to Figure 1.

Subunit	Chain	Total residues	Modeled residues	% Modeled	Qscore	Notes
**NDUFV1**	F	444	10-437	96.4	0.63 (0.54)	FMN, 4Fe4S
**NDUFV2**	E	217	5-216	97.7	0.61 (0.51)	2Fe2S
**NDUFS1**	G	704	7-692 (6-693)	97.7 (97.4)	0.65 (0.55)	2Fe2S, 2 x 4Fe4S
**NDUFS2**	D	430	1-38, 46-54, 61-430 (1-39, 46-54, 60-430)	97.4 (97.0)	0.69 (0.58)	Dimethyl-Arg85
**NDUFS3**	C	228	7-213 (8-214)	90.8 (90.8)	0.69 (0.59)	
**NDUFS7**	B	189	35-189	82.0	0.68 (0.58)	4Fe4S
**NDUFS8**	I	178	1-178	100	0.69 (0.59)	2 x 4Fe4S<br>no sidechains 8-14
**ND1**	H	318	1-60, 67-206, 214-318 (1-203, 214-318)	96.9 (95.9)	0.66 (0.55)	N-formyl
**ND2**	N	345	1-344	99.7	0.67 (0.56)	N-formyl
**ND3**	A	115	1-26, 49-115 (1-25, 51-115)	78.2 (80.9)	0.65 (0.52)	N-formyl
**ND4**	M	459	1-459	100	0.67 (0.56)	N-formyl
**ND4L**	K	98	1-98	100	0.66 (0.56)	N-formyl
**ND5**	L	607	1-606	99.8	0.64 (0.53)	N-formyl
**ND6**	J	172	1-171	99.4	0.62 (0.50)	N-formyl<br>no sidechains 108-112
**NDUFV3**	s	69	30-68	56.5	0.62 (0.53)	
**NDUFS4**	Q	133	9-133	94.0	0.67 (0.57)	
**NDUFS5**	e	105	1-104	99.0	0.66 (0.55)	2 x Cys-Cys (no Cys-Cys)
**NDUFS6**	R	96	1-94	97.9	0.68 (0.57)	Zn^{2+}
**NDUFA1**	a	70	1-68	97.2	0.67 (0.55)	
**NDUFA2**	S	98	14-95	83.7	0.58 (0.48)	
**NDUFA3**	b	83	2-83 (1-83)	100 (98.8)	0.62 (0.51)	
**NDUFA5**	V	115	4-115	97.4	0.62 (0.53)	
**NDUFA6**	W	130	17-130	87.7	0.63 (0.52)	
**NDUFA7**	r	112	1-73, 90-112	85.7	0.66 (0.55)	N-acetyl
**NDUFA8**	X	171	1-171	100	0.65 (0.55)	(2 x Cys-Cys)
**NDUFA9**	P	342	1-185, 200-249, 280-323, 334-342 (1-185, 199-249, 279-323, 334-342)	85.1 (84.2)	0.62 (0.53)	NADPH
**NDUFA10**	O	320	2-320 (1-320)	100 (99.7)	0.65 (0.54)	ATP
**NDUFA11**	Y	140	1-140	100	0.63 (0.50)	2 x Cys-Cys (1 x Cys-Cys)
**NDUFA12**	q	145	2-144 (1-144)	99.3 (98.6)	0.68 (0.56)	N-acetyl
**NDUFA13**	Z	143	5-143	97.2	0.65 (0.55)	
**NDUFAB1α**	T	88	11-82 (10-82)	83.0 (81.8)	0.47 (0.37)	4’-phosphopantethine +<br>3-hydroxyundecanoate
**NDUFAB1β**	U	88	1-88	100	0.59 (0.48)	
**NDUFB1**	f	56	4-54 (5-54)	89.3 (91.1)	0.61 (0.50)	
**NDUFB2**	j	72	7-68 (6-68)	87.5 (86.1)	0.60 (0.48)	
**NDUFB3**	k	103	18-94 (17-94)	75.7 (74.8)	0.58 (0.50)	
**NDUFB4**	m	128	4-128	97.7	0.63 (0.52)	
**NDUFB5**	h	143	7-143	95.8	0.67 (0.56)	
**NDUFB6**	i	127	2-35, 64-124 (1-35, 64-124)	75.6 (74.8)	0.60 (0.49)	N-acetyl
**NDUFB7**	o	136	2-115 (2-112)	81.6 (83.8)	0.56 (0.47)	
**NDUFB8**	l	157	2-157	99.4	0.65 (0.54)	
**NDUFB9**	n	178	1-176	98.9	0.64 (0.52)	
**NDUFB10**	p	175	4-171	96.0	0.63 (0.52)	2 x Cys-Cys
**NDUFB11**	g	122	19-120 (21-121)	82.7 (83.6)	0.64 (0.54)	
**NDUFC1**	c	49	2-47	93.9	0.61 (0.51)	
**NDUFC2**	d	120	1-119	99.1	0.66 (0.56)	
