# Supplementary material for: Structural basis for a complex I mutation that blocks pathological ROS production
Source: Nat Commun. 2021 Jan 29;12:707. doi: 10.1038/s41467-021-20942-w (PMC7846746; doi:10.1038/s41467-021-20942-w)
Supplement: Supplementary file 2 — Description of Additional Supplementary Files [file 41467_2021_20942_MOESM2_ESM.pdf]

Description of Additional Supplementary Files

Supplementary Movie 1: This movie shows the effects of the ND6-P25L mutation on the conformational changes of subunit ND6 during the A/D transition of complex I.
