# Supplementary material for: Structural basis for a complex I mutation that blocks pathological ROS production
Source: Nat Commun. 2021 Jan 29;12:707. doi: 10.1038/s41467-021-20942-w (PMC7846746; doi:10.1038/s41467-021-20942-w)
Supplement: Supplementary file 4 — Reporting Summary [file 41467_2021_20942_MOESM4_ESM.pdf]

nature research

Corresponding author(s): Michael P. Murphy & Judy Hirst

Last updated by author(s): Dec 12, 2020

Reporting Summary

Nature Research wishes to improve the reproducibility of the work that we publish. This form provides structure for consistency and transparency in reporting. For further information on Nature Research policies, see our Editorial Policies and the Editorial Policy Checklist.

Statistics

For all statistical analyses, confirm that the following items are present in the figure legend, table legend, main text, or Methods section.

n/a	Confirmed	
☐	☒	The exact sample size (n) for each experimental group/condition, given as a discrete number and unit of measurement
☐	☒	A statement on whether measurements were taken from distinct samples or whether the same sample was measured repeatedly
☐	☒	The statistical test(s) used AND whether they are one- or two-sided<br>*Only common tests should be described solely by name; describe more complex techniques in the Methods section.*
☐	☒	A description of all covariates tested
☐	☒	A description of any assumptions or corrections, such as tests of normality and adjustment for multiple comparisons
☐	☒	A full description of the statistical parameters including central tendency (e.g. means) or other basic estimates (e.g. regression coefficient) AND variation (e.g. standard deviation) or associated estimates of uncertainty (e.g. confidence intervals)
☐	☒	For null hypothesis testing, the test statistic (e.g. F, t, r) with confidence intervals, effect sizes, degrees of freedom and P value noted<br>*Give P values as exact values whenever suitable.*
☐	☒	For Bayesian analysis, information on the choice of priors and Markov chain Monte Carlo settings
☒	☐	For hierarchical and complex designs, identification of the appropriate level for tests and full reporting of outcomes
☒	☐	Estimates of effect sizes (e.g. Cohen's d, Pearson's r), indicating how they were calculated

Our web collection on statistics for biologists contains articles on many of the points above.

Software and code

Policy information about availability of computer code

Data collection	CryoEM data collection: FEI EPU 1.9<br><br>Mass spectrometry data collection: MassLynx 4.1 software, Labsolutions software.
Data analysis	CryoEM data processing: RELION-3.0, RELION-3.1, Motioncor2,Gctf-1.18, UCSF Chimera-1 13.1<br><br>Complex I model building:Coot 0.9-pre, Phenix-1.16-3549, ISOLDE 1.0, SCIPION 1.2, MolProbity 4.4, EMRinger-v1.0.0, UCSF Chimera and PyMol 2.3.5.<br><br>Mass spectrometry data analysis: MassLynx 4.1 software, Labsolutions 5.82 software.<br><br>Figure preparation and statistical analysis: GraphPad Prism Version 8<br><br>Software for Langendorff heart perfusion function: AD Instruments Chart (v 7.0), Data Analysis: Graphpad Prism (v 8.3), Microsoft Excel (v.16.16.15).

For manuscripts utilizing custom algorithms or software that are central to the research but not yet described in published literature, software must be made available to editors and reviewers. We strongly encourage code deposition in a community repository (e.g. GitHub). See the Nature Research guidelines for submitting code & software for further information.

Data

Policy information about availability of data

All manuscripts must include a data availability statement. This statement should provide the following information, where applicable:

- Accession codes, unique identifiers, or web links for publicly available datasets
- A list of figures that have associated raw data
- A description of any restrictions on data availability

The electron density maps were deposited into EMDB and the models were deposited into PDB. EMD-11811, PDB ID: 7AK6 (ND6-P25L) and EMD-11810, PDB ID: 7AK5 (deactive state). All data are available in the manuscript or the supplementary materials. The structure coordinates and Cryo-EM maps generated during this study are available at the RCSB PDB with accession codes: EMD-11811, PDB ID: 7AK6 (ND6-P25L) and EMD-11810, PDB ID: 7AK5 (deactive state). Source data for Figs. 2–4 and S5 are provided with this paper.

Field-specific reporting

Please select the one below that is the best fit for your research. If you are not sure, read the appropriate sections before making your selection.

- [x] Life sciences
- [ ] Behavioural & social sciences
- [ ] Ecological, evolutionary & environmental sciences

For a reference copy of the document with all sections, see nature.com/documents/nr-reporting-summary-flat.pdf

Life sciences study design

All studies must disclose on these points even when the disclosure is negative.

Sample size	CryoEM - a single data collection was used for each of the ND6 P35L and WT deactive structures: starting particles were 42,622 and 148,440 respectively, filtered by 2D and 3D classification, and final particles were 25,629 and 50,184 respectively. For biochemical analyses samples sizes of n=3 or larger (as specified) were chosen. Replicates are specified as biological or technical as appropriate. Specific sample size calculations were not performed and sample sizes used are standard for these fields and sufficient to enable robust statistical significance to be determined.
Data exclusions	CryoEM - 2D images were picked automatically and classified, then data for non-protein or damaged protein images were removed (see Methods). No data were excluded from other experiments.
Replication	The acquired data were highly consistent and reproducible between different experimental days and biological samples. The specific number of replicates are listed in the figure legends for each experiment.
Randomization	Animals were randomly assigned in experimental or control groups in all experiments.
Blinding	Infarct size measurements and mass spectrometry were carried out in a randomized and blinded fashion. Blinding was not appropriate fro all other experiments.

Reporting for specific materials, systems and methods

We require information from authors about some types of materials, experimental systems and methods used in many studies. Here, indicate whether each material, system or method listed is relevant to your study. If you are not sure if a list item applies to your research, read the appropriate section before selecting a response.

Materials & experimental systems

n/a	Involved in the study	
x		Antibodies
x		Eukaryotic cell lines
x		Palaeontology and archaeology
	x	Animals and other organisms
x		Human research participants
x		Clinical data
x		Dual use research of concern

Methods

n/a	Involved in the study	
x		ChIP-seq
x		Flow cytometry
x		MRI-based neuroimaging

Animals and other organisms

Policy information about studies involving animals; ARRIVE guidelines recommended for reporting animal research

Laboratory animals	Mice wild-type and ND6-P25L (both C57BL/6J background) were used in this study. Mice (both male and female as specified in the individual section) were between 8-22 weeks of age. Mice were maintained with a 12h:12h light: dark cycle, a room temperature of

	19˚C to 22˚C, relative humidity 55% ± 10%, and with ad lib food and water.
Wild animals	no wild animals were used in the study.
Field-collected samples	no field collected samples were used in the study.
Ethics oversight	All procedures were carried out in accordance with the UK Animals (Scientific Procedures) Act, 1986. Experiments at the University of Cambridge were carried out under PPL: P6C97520A, approved by the local ethics committees of the MRC Laboratory of Molecular Biology and the University of Cambridge and by the UK Home Office. Experiments at Queen Mary University of London were carried out under licence: PPL PB137135C, approved by the local ethics committee of Queen Mary University of London and by the UK Home Office.

Note that full information on the approval of the study protocol must also be provided in the manuscript.
